# Zyxin is important for the stability and function of podocytes, especially during mechanical stretch

**DOI:** 10.1038/s42003-024-06125-5

**Published:** 2024-04-11

**Authors:** Felix Kliewe, Florian Siegerist, Elke Hammer, Jaafar Al-Hasani, Theodor Rolf Jakob Amling, Jonas Zeno Eddy Hollemann, Maximilian Schindler, Vedran Drenic, Stefan Simm, Kerstin Amann, Christoph Daniel, Maja Lindenmeyer, Markus Hecker, Uwe Völker, Nicole Endlich

**Affiliations:** 1 Department of Anatomy and Cell Biology, University Medicine Greifswald, Greifswald, Germany; 2 Interfaculty Institute for Genetics and Functional Genomics, University Medicine Greifswald, Greifswald, Germany; 3 Department of Cardiovascular Physiology, Heidelberg University, Heidelberg, Germany; 4 NIPOKA GmbH, Center of High-End Imaging, Greifswald, Germany; 5 Institute of Bioinformatics, University Medicine Greifswald, Greifswald, Germany; 6 Department of Nephropathology; Friedrich-Alexander University (FAU) Erlangen-Nuremberg, Erlangen, Germany; 7 III. Department of Medicine, University Medical Center Hamburg-Eppendorf, Hamburg, Germany; 8 Hamburg Center for Kidney Health (HCKH), University Medical Center Hamburg-Eppendorf, Hamburg, Germany

**Keywords:** Cell biology, Kidney, Kidney diseases, Cytoskeleton, Mechanisms of disease

## Abstract

Podocyte detachment due to mechanical stress is a common issue in hypertension-induced kidney disease. This study highlights the role of zyxin for podocyte stability and function. We have found that zyxin is significantly up-regulated in podocytes after mechanical stretch and relocalizes from focal adhesions to actin filaments. In zyxin knockout podocytes, we found that the loss of zyxin reduced the expression of vinculin and VASP as well as the expression of matrix proteins, such as fibronectin. This suggests that zyxin is a central player in the translation of mechanical forces in podocytes. In vivo, zyxin is highly up-regulated in patients suffering from diabetic nephropathy and in hypertensive DOCA-salt treated mice. Furthermore, zyxin loss in mice resulted in proteinuria and effacement of podocyte foot processes that was measured by super resolution microscopy. This highlights the essential role of zyxin for podocyte maintenance in vitro and in vivo, especially under mechanical stretch.

## Abstract (web summary)

This study highlights the essential role of zyxin for podocytes. Zyxin loss in vitro reduced the expression of vinculin, VASP and matrix proteins. In mice, the knockout of zyxin resulted in proteinuria and effacement of podocyte foot processes.

## Introduction

More than 10% of the population worldwide suffer from chronic kidney disease (CKD)^1^. Diabetes and hypertension, which are the major causes for the development of end-stage kidney disease (ESKD)^2,3^, are often associated with glomerular hypertension. Glomerular hypertension is assumed to damage specifically podocytes, a terminally differentiated epithelial cell type in the glomerulus with extended major processes and thin, interdigitating foot processes (FP) attached to the glomerular basement membrane (GBM). This complex 3D morphology of podocytes is highly dependent on an intact actin cytoskeleton that is mainly located subcortical and in podocyte FP. Alterations of the actin cytoskeleton and actin-associated proteins often lead to a broadening of FP and a detachment of podocytes, often resulting in the loss of the size-selectivity of the glomerular filtration barrier^4–7^. Since podocyte detachment cannot be compensated, the loss of podocytes is terminal.

To prevent podocyte detachment and damage induced by glomerular hypertension, knowledge about the responsible mechanosensors and mechanotransducers is essential. In the past, our group has revealed that cultured podocytes are mechanosensitive and change their actin cytoskeleton as well as the gene expression upon mechanical stress and flow-induced shear stress^8,9^. We found that mechanically stressed podocytes reorganize their actin cytoskeleton from transversal stress fibers into radially oriented actin filaments converging into a so called *actin rich center* (ARC)^9^. However, the mechanosensor or mechanotransducer, which is responsible for such an actin reorganization, is still unknown.

There are different ways in which cells sense physical forces^10,11^. It is postulated that ion channels become activated by mechanical stretch allowing ion influx that triggers signaling cascades^11–13^. Another mechanism is that focal adhesions (FAs) and the actin cytoskeleton itself activate specific pathways in response to physical forces^14–16^. Based on this, FA proteins are predisposed to be the linker between integrins and the actin cytoskeleton for such a mechanotransduction^17–24^.

Zyxin is an important FA and actin-associated protein, which has already been described to play an important role in the cellular response to mechanical forces^25–27^. In this regard, zyxin has been implicated in facilitating actin filament assembly and stabilization of actin stress fibers, thereby propagating force transmission from the extracellular matrix to the nucleus^14,28–33^. Zyxin is a zinc-binding phosphoprotein with a N-terminal proline-rich domain and three LIM domains proximal to the C-terminus^34,35^. The N-terminus of zyxin has been reported to bind the actin filament crosslinker α-actinin, the actin assembly modulator Ena/VASP, the cytoskeletal protein LIM and SH3 domain protein 1 (LASP-1)^36–39^ as well as the stretch-sensitive protein p130Cas^40^. In particular, the LIM domains of zyxin are essential for actin binding and stress fiber localization in cells exposed to uniaxial stretch. This might be sufficient for force-induced accumulation of zyxin during cell migration^41–43^ .

In vivo, zyxin was described to protect from hypertension-induced cardiac dysfunction^44^ as well as being able to function a mechanosensor^26^.

The present study investigated the role of zyxin in mechanically stretched podocytes, a model for glomerular hypertension. Here we describe that zyxin is essential for the adhesion of the postmitotic cell type, the podocytes, in vitro during mechanical stretch as well as in vivo for the maintenance of the kidney filtration.

## Results

### Zyxin is localized at focal adhesions and actin fibers in cultured murine podocytes

**Fig. 1:**
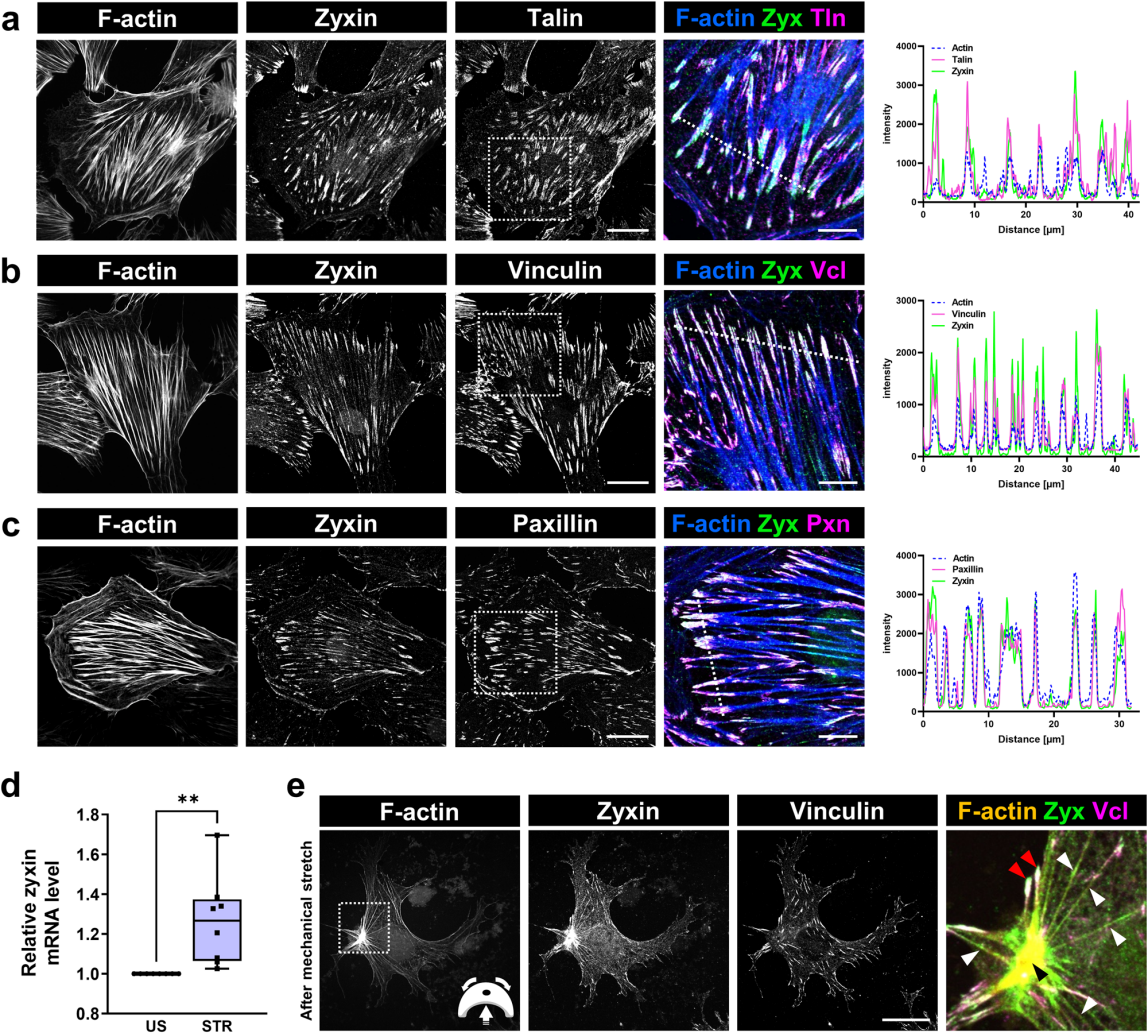
Expression and colocalization of F-actin and the focal adhesion proteins talin, vinculin and paxillin with zyxin in cultured podocytes. Immunofluorescence analysis of cultured podocytes showed a colocalization of zyxin (green), F-actin (blue) and (**a**) talin, (**b**) vinculin and (**c**) paxillin (shown in magenta). White dotted box shows area of magnification. White dotted line marks the plot profile, which confirms the colocalization of zyxin with the other focal adhesion proteins. The scale bar represents 25 µm and 10 µm (magnification). **d** qRT-PCR based relative mRNA level of zyxin in unstretched and stretched podocytes (n = 8; *p* = 0.0046). **e** Mechanically stretched podocytes reorganized their cytoskeleton and formed actin-rich centers (ARCs), which showed an accumulation of zyxin (green) (marked by black arrowhead). The white arrowheads show the binding of zyxin to the actin fibers (orange) caused by mechanical stretch. In contrast, vinculin (magenta) is only located at the end of the F-actin (marked by red arrowheads). The scale bar represents 25 μm.

To study the expression of zyxin in cultured mouse podocytes, cells were stained with antibodies against zyxin and the focal adhesion proteins talin-1, vinculin and paxillin (Fig. 1a–c). By using confocal laser scanning microscopy, we found that zyxin is colocalized with focal adhesion proteins as well as at actin fibers, as indicated by the plot profile intensity of multiple fluorescence images (Fig. 1a–c, last panels).

### Mechanical stretch regulates zyxin expression and localization

To find out whether mechanical stretch regulates the expression of zyxin, podocytes were cultured on flexible silicone membranes and stretched for 3 days (0.5 Hz and 5% elongation) as described previously^9^. The expression of zyxin was analyzed in stretched (STR) and unstretched (US) podocytes by immunofluorescence staining and qRT-PCR (Fig. 1d, e). We found a slight, but significant increase of the *Zyx* mRNA level due to mechanical stretch (Fig. 1d). Furthermore, we observed a reorganization of the podocyte actin cytoskeleton from transversal stress fibers into radial stress fibers due to mechanical stretch (Fig. 1e), as we have already described^9^. We have found that mechanical stretch additionally induced increased zyxin localization along actin filaments as well as in the actin rich center (ARC) (Fig. 1e). This re-localization was also observed in mechanically stretched primary podocytes. (Fig. S1a).

### Zyxin knockout podocytes show reduced cell adhesion and higher cell loss after mechanical stretch

**Fig. 2:**
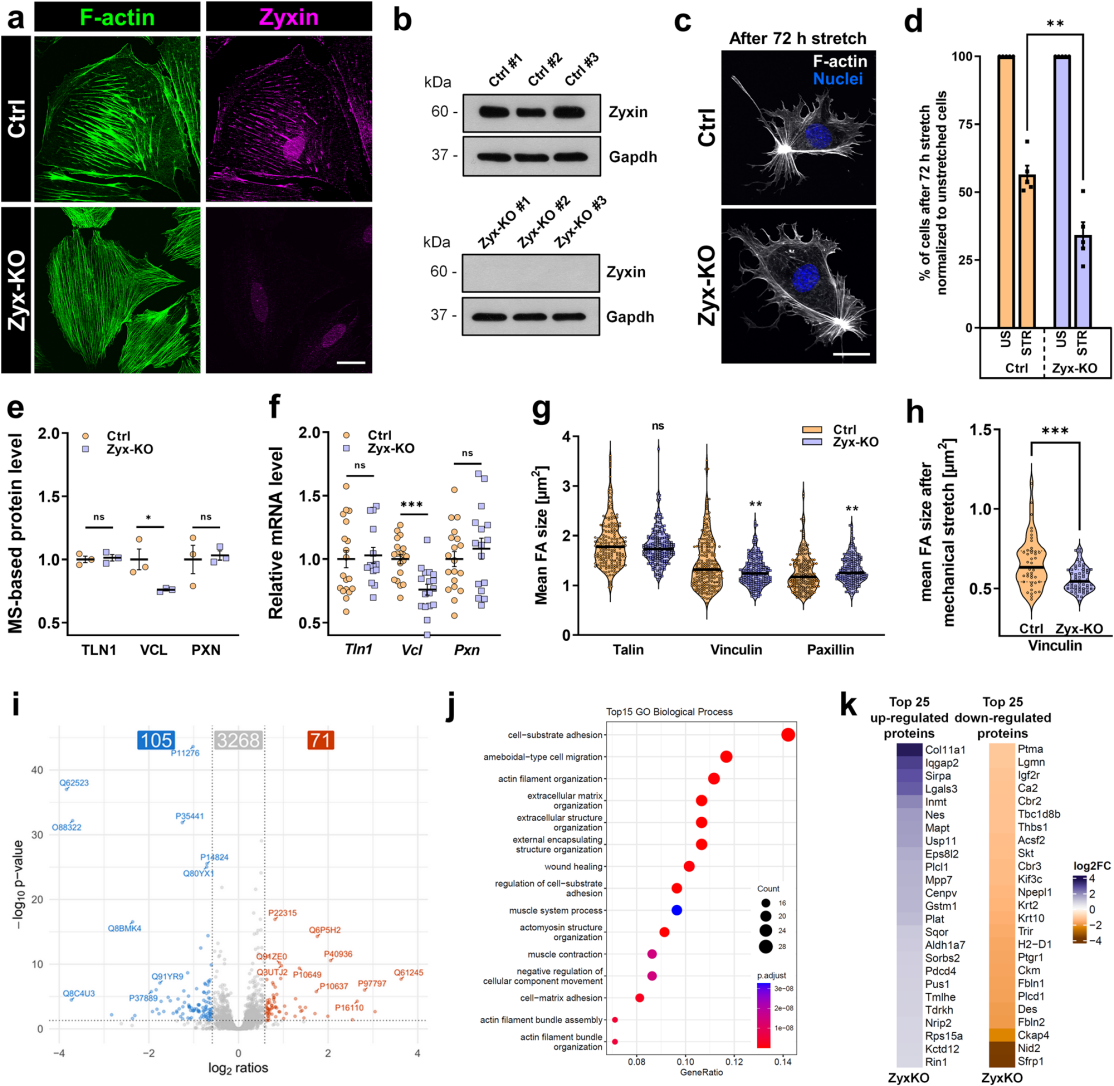
Verification and proteome analysis of zyxin KO podocytes. **a** Immunofluorescence staining confirmed the loss of zyxin in Zyx-KO podocytes. Zyxin is shown in magenta; F-actin in green. Scale bar represents 25 μm. **b** Western blot verified the complete loss of zyxin in Zyx-KO podocytes. Gapdh served as a loading control. **c** Zyx-KO podocytes showed no reduced reorganization of the F-actin (white) and no reduction in formation of actin rich centers. The nuclei shown in blue. Scale bar represents 25 μm. **d** Zyx-KO podocytes showed a significant loss of cells compared to the control (Ctrl) after 3 days mechanical stretch (n = 5). **e** LC-MS/MS based relative protein level of talin (TLN1), vinculin (VCL) and paxillin (PXN) in Zyx-KO and control podocytes (n = 3). **f** The mRNA expression of the focal adhesion proteins talin, vinculin and paxillin in Zyx-KO and control podocytes (Ctrl) was quantified by qRT-PCR (n ≥ 14). qRT-PCR experiments were normalized to Ctrl, *Gapdh* served as a reference. **g** IF pictures were randomly analysed from four independent experiments with the “Focal Contact Segmentation and Analysis Tool” and revealed a significant reduction of the single FA area size (given in µm^2^) of vinculin in Zyx-KO podocytes compared to Ctrl (n ≥ 173 cells). **h** Mean FA size of vinculin after mechanical stretch in Ctrl and Zyx-KO podocytes (n ≥ 44 cells). **i** Volcano plot displaying proteins with significantly changed abundance in Zyx-KO podocytes in comparison to Ctrl podocytes (105 down-, 71 up-regulated proteins). Blue: down-regulated compared to controls; red: up-regulated compared to controls. Given are proteins with a fold-change ≥1.5 and *p* value ≤ 0.05. **j** Top 15 GO (Gene Ontology) clusters in Zyx-KO podocytes, which were significant down-regulated compared to Ctrl. **k** Top 25 significantly up- and down-regulated proteins in Zyx-KO podocytes. Data are represented as log2 fold change, a *p* value < 0.05 was considered as significant. Blue: up-regulated compared to controls; orange: down-regulated compared to controls. Data are presented as means ± SEM (**d**–**f**) or violin plot (**g**, **h**); * *p* < 0.05; ** *p* < 0.01; *** *p* < 0.001; ns, not significant.

To study the role of zyxin in cultured podocytes that were exposed to mechanical stress for 3 days, we generated a zyxin knockout podocyte cell line (Zyx-KO) via the CRISPR/Cas9 technique and stretched Zyx-KO and control podocytes (Ctrl) under the same conditions. The knockout was confirmed by immunofluorescence staining and Western blot analysis (Fig. 2a, b).

After mechanical stretch, Zyx-KO podocytes showed a higher cell loss compared to the Ctrl (-40 ± 10%) (Fig. 2d). However, we observed that mechanical stretch had no effect on the stretch-induced reorganization of the F-actin cytoskeleton and the formation of ARCs of the remaining Zyx-KO podocytes (Fig. 2c).

### Zyxin regulates the expression of distinct focal adhesions

To investigate whether the reduced cell adhesion under mechanical stress is due to a loss of focal adhesion proteins, we determined the expression levels of the typical focal adhesion (FA) proteins talin, vinculin and paxillin in Zyx-KO podocytes by proteomics, qRT-PCR as well as immunocytochemistry analysis. Zyx-KO podocytes showed a significant decrease of vinculin protein (-23%) as well as mRNA level (-25%) compared to Ctrl (Fig. 2e, f). Interestingly, the expression of talin and paxillin was not significantly affected by the zyxin knockout (Fig. 2e, f). Furthermore, immunocytochemistry analysis showed that the loss of zyxin significantly influenced the size of FAs (Fig. 2g). The quantification revealed that the single FA area size was significantly reduced for vinculin (mean FA size in µm^2^: Ctrl: 1.32; Zyx-KO: 1.24). The FA size for talin was unchanged (Ctrl: 1.78 µm^2^; Zyx-KO: 1.73 µm^2^) and paxillin-positive focal adhesions were even enlarged (Ctrl: 1.17 µm^2^; Zyx-KO: 1.26 µm^2^). Interestingly, the decrease in the mean FA size of vinculin is more pronounced under mechanical stretch in comparison to the unstretched conditions (Fig. 2h and Fig. S1b).

To find out which other proteins are regulated by zyxin, we performed proteomic profiling of three independent Ctrls (control podocytes) and three Zyx-KO podocyte clones by LC-MS/MS (Supplementary Data 1). We detected 71 significant up- and 105 down-regulated proteins in the Zyx-KO podocytes compared to the control group (*p* < 0.05 and fold change >1.5) (Fig. 2i). Figure 2k shows the top 25 up- and down-regulated proteins. It was remarkable that in the Zyx-KO podocytes the Gene Ontology clusters were strongly down-regulated, which are related to cell adhesion or actin filament organization (Fig. 2j).

### Zyxin regulates the expression of VASP and actin filament organization proteins in podocytes

**Fig. 3:**
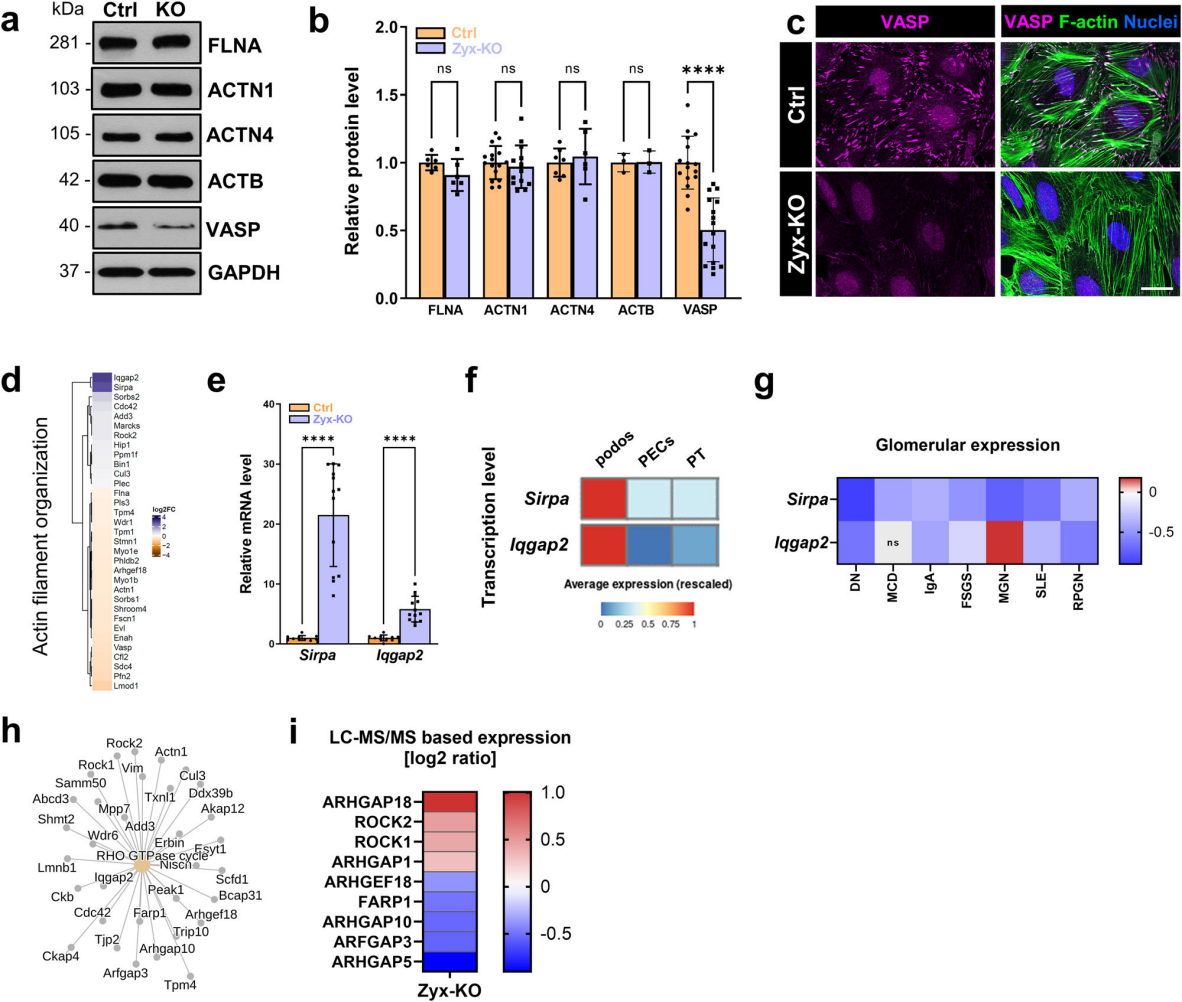
Zyxin regulates the expression of VASP, *Sirpa* and *Iqgap2* in podocytes. **a** Western blot analyses of different actin-associated proteins in Zyx-KO podocytes. **b** Western blot quantification of Zyx-KO podocytes showed a significant decrease of the VASP protein abundance compared to the control (Ctrl) (n = 16). However, the expression of FLNA, ACTN1, ACTN4 and ACTB remained unchanged (n ≥ 3). GAPDH served as a loading control. Data are presented as means ± SD. **c** Immunofluorescence staining of VASP (magenta) and F-actin (green) showed decreased signal of VASP in Zyx-KO podocytes. The scale bar represents 25 μm. **d** Heatmap of regulated “actin filament organization” proteins (GO term) in Zyx-KO podocytes. Data are represented as log2 fold change, a *p* value < 0.05 was considered as significant. Blue: up-regulated; orange: down-regulated compared to controls. **e** mRNA expression of *Sirpa* and *Iqgap2* normalized to Ctrl and *Gapdh*. **f** Database analysis of *Sirpa* and *Iqgap2* in podos (podocytes), PECs (parietal epithelial cells) and PT (proximal tubule cells) using the KidneyCellExplorer (Ransick et al. ^45^) based on a single‐cell RNA sequencing data set of murine kidneys. (g) mRNA expression level of microdissected glomeruli from renal biopsies of human patients suffering from diabetic nephropathy (DN; n = 14), minimal change disease (MCD; n = 14), IgA nephropathy (IgA; n = 27), focal segmental glomerulosclerosis (FSGS; n = 23), membranous nephropathy (MGN; n = 21), lupus nephritis (SLE; n = 32) and rapidly progressive glomerulonephritis (RPGN, n = 23) compared with healthy living donors (LD; n = 41). Data are represented as log2 fold change, a q-value < 0.05 was considered as significant. Red: up-regulated; blue: down-regulated compared to LD. **h** Gene set enrichment analysis reveals an increased regulation of proteins of the “Rho GTPase cycle” in Zyx-KO podocytes. **i** LC-MS/MS based relative protein level of different proteins of the Rho GTPase cycle. Data are represented as log2 fold change, a *p* value < 0.05 was considered as significant (n = 3). Red: up-regulated; blue: down-regulated compared to controls. Data are presented as means ± SD (**b**, **e**); **** *p* < 0.0001; ns, not significant.

To study the influence of zyxin on the expression of the zyxin-binding protein VASP, we performed immunostainings as well as Western blot analyses (Fig. 3a–c). Zyx-KO podocytes showed a significant decrease of the protein level of VASP to 51 ± 22% compared to the Ctrl (Fig. 3a, b). The expression of other actin-associated proteins such as filamin A, α-actinin-1 or α-actinin-4 was not affected by the loss of zyxin (Fig. 3a, b). Furthermore, immunostainings showed that the recruitment of VASP to the actin filaments seemed to be disrupted in Zyx-KO podocytes (Fig. 3c). This suggests that VASP is recruited to actin stress fibers in a zyxin-dependent manner. In addition to VASP, other proteins of the zyxin interaction network were also significantly affected in Zyx-KO podocytes (Fig. S2a, b).

Proteomic data further showed a significant regulation of the Gene Ontology cluster “actin filament organization” in Zyx-KO podocytes (Fig. 3d). Especially, the protein levels of SIRPA (tyrosine-protein phosphatase non-receptor type substrate 1) and IQGAP2 (Ras GTPase-activating-like protein) were significantly increased (SIRPA: 7.03-fold up-regulated; IQGAP2: 8.26-fold up-regulated). We confirmed this by qRT-PCR and found that mRNA levels of *Sirpa* and *Iqgap2* were significantly increased in Zyx-KO podocytes compared to Ctrl (*Sirpa*: 21.5-fold up-regulated; *Iqgap2*: 5.8-fold up-regulated) (Fig. 3e). Transcriptional expression levels based on single‐cell RNA sequencing data^45^ revealed that *Sirpa* and *Iqgap2* are both highly expressed in podocytes (Fig. 3f and Fig. S3). Looking at the expression levels of these proteins in patients suffering from diabetic nephropathy (DN), we found a down-regulation of the expression (log2 fold change: *SIRPA*: -0.902; *IQGAP2*: -0.578) (Fig. 3g). Moreover, we found that the loss of zyxin did not only leads to an up-regulation of Rho GTPase activating protein IQGAP2, but also affected the expression of other Rho GTPase-associated proteins like guanine nucleotide exchange factors (GEFs) and GTPase-activating proteins (GAPs). A high number of Arhgap and Arhgef proteins were also regulated by zyxin in cultured podocytes (Fig. 3h, i).

### Zyxin knockout podocytes show reduced expression of extracellular matrix proteins and diminished cell spreading

**Fig. 4:**
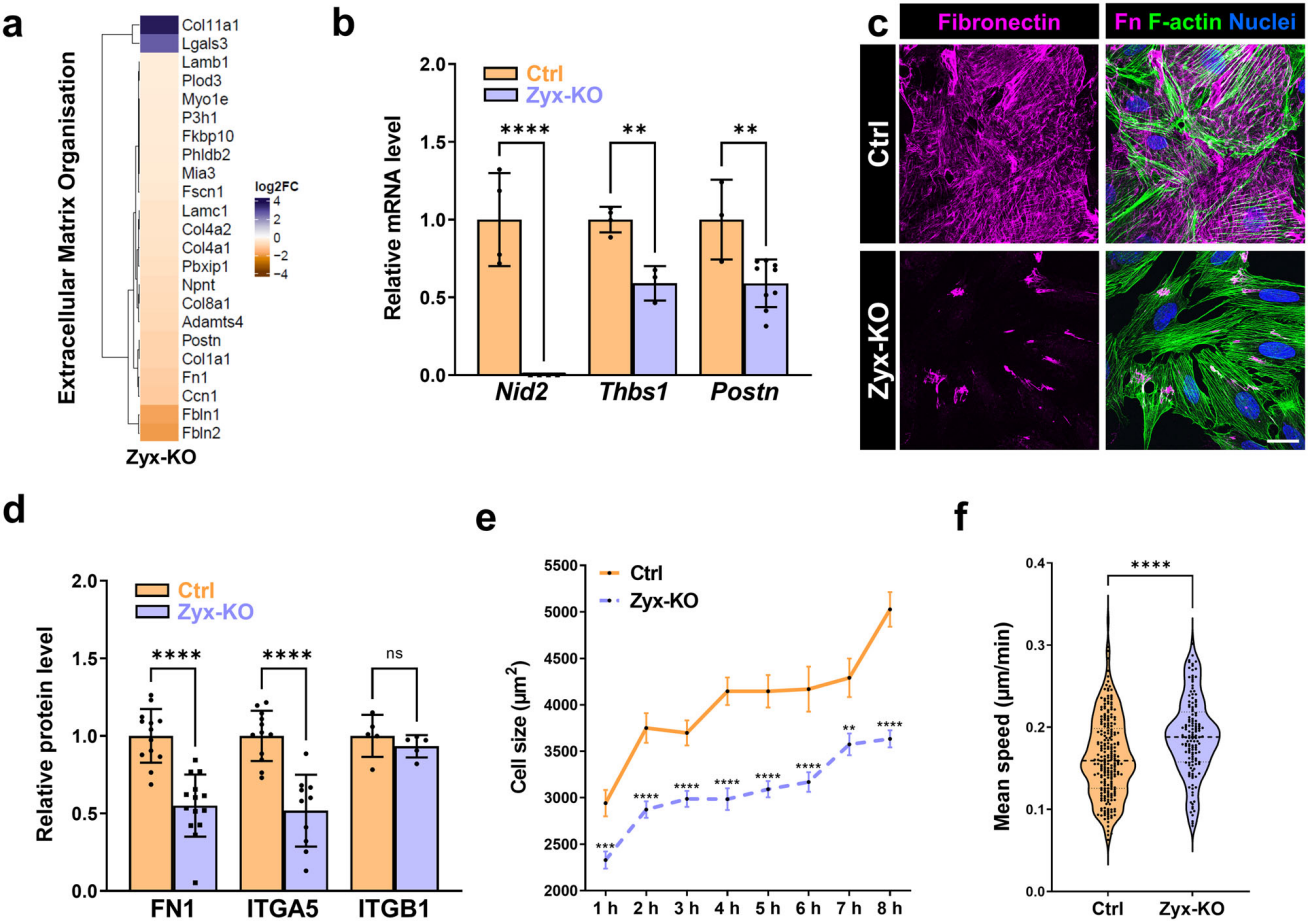
Zyx-KO podocytes showed a reduced expression of extracellular matrix proteins. **a** Ratios of regulated “extracellular matrix organisation” proteins (GO term) in Zyx-KO podocytes. Data are represented as log2 fold change, a *p* value < 0.05 was considered as significant. Blue: up-regulated; orange: down-regulated compared to controls. **b** The mRNA expression of nidogen-2 (*Nid2*), thrombospondin-1 (*Thbs1*) and periostin (*Postn*) in Zyx-KO and control podocytes (Ctrl) was quantified by qRT-PCR (n ≥ 3). qRT-PCR experiments were normalized to the Ctrl, *Gapdh* served as a reference. **c** Immunostaining of fibronectin (shown in magenta) of Zyx-KO and control (Ctrl) podocytes. Scale bar represents 25 μm. **d** Western blot analysis showed decreased fibronectin protein level compared to control podocytes (n = 13). In addition, the protein levels of ITGA5 and ITGB1 are shown (n ≥ 5). **e** Cell sizes of Zyx-KO and control podocytes were quantified at different time points (1 –8 h) and are given as µm^2^ (n = 100 cells per time point). **f** Quantification of migration (data represents mean speed [µm/min]) from a time-lapse movie over 20 h. Each dot represents one individual cell (233 Ctrl and 141 Zyx-KO podocytes were analyzed). ** *p* < 0.01; *** *p* < 0.001; **** *p* < 0.0001; ns, not significant. Data are presented as means ± SD (**b**; **d**) or means ± SEM (**e**).

Proteomic data impressively showed that extracellular matrix (ECM) proteins are significantly down-regulated in Zyx-KO podocytes (Fig. 4a). Therefore, we analyzed the mRNA levels of specific ECM proteins by qRT-PCR. We have found that the mRNA levels of nidogen-2 (*Nid2*), thrombospondin-1 (*Thbs1*) and periostin (*Postn*) were significantly reduced in Zyx-KO podocytes (Fig. 4b). To explore whether the loss of zyxin also influences the expression of fibronectin, we performed immunostainings and Western blot analyses of Zyx-KO and Ctrl podocytes (Fig. 4c, d). The protein level of fibronectin was significantly reduced by 45% in Zyx-KO podocytes compared with control podocytes (Fig. 4d). This was also confirmed by immunofluorescence analysis, which revealed a marked decrease of fibronectin fibrils in the Zyx-KO podocytes (Fig. 4c).

Additionally, we found that the expression of the specific fibronectin receptor integrin α5 was significantly reduced (-48%) in Zyx-KO podocytes. In contrast, the protein level of integrin β1 did not change (Fig. 4d). Since zyxin deficiency strongly reduced the expression of ECM proteins, we wanted to investigate the role of zyxin in cell spreading. For this, both Ctrl and Zyx-KO podocytes were trypsinized, seeded on glass cover slips and incubated for 1–8 h. After fixation at different time points, the cell size of the podocytes was quantified (Fig. 4e). We found that Zyx-KO podocytes showed a reduced spreading and cell size in the initial phase of adhesion compared to Ctrl (Fig. 4e). Furthermore, the migration speed and distance increased significantly in Zyx-KO podocytes in contrast to the Ctrl (Fig. 4f and Fig. S1c).

### The loss of zyxin reduces the number and length of filopodia in cultured podocytes due to a down-regulation of fascin-1

**Fig. 5:**
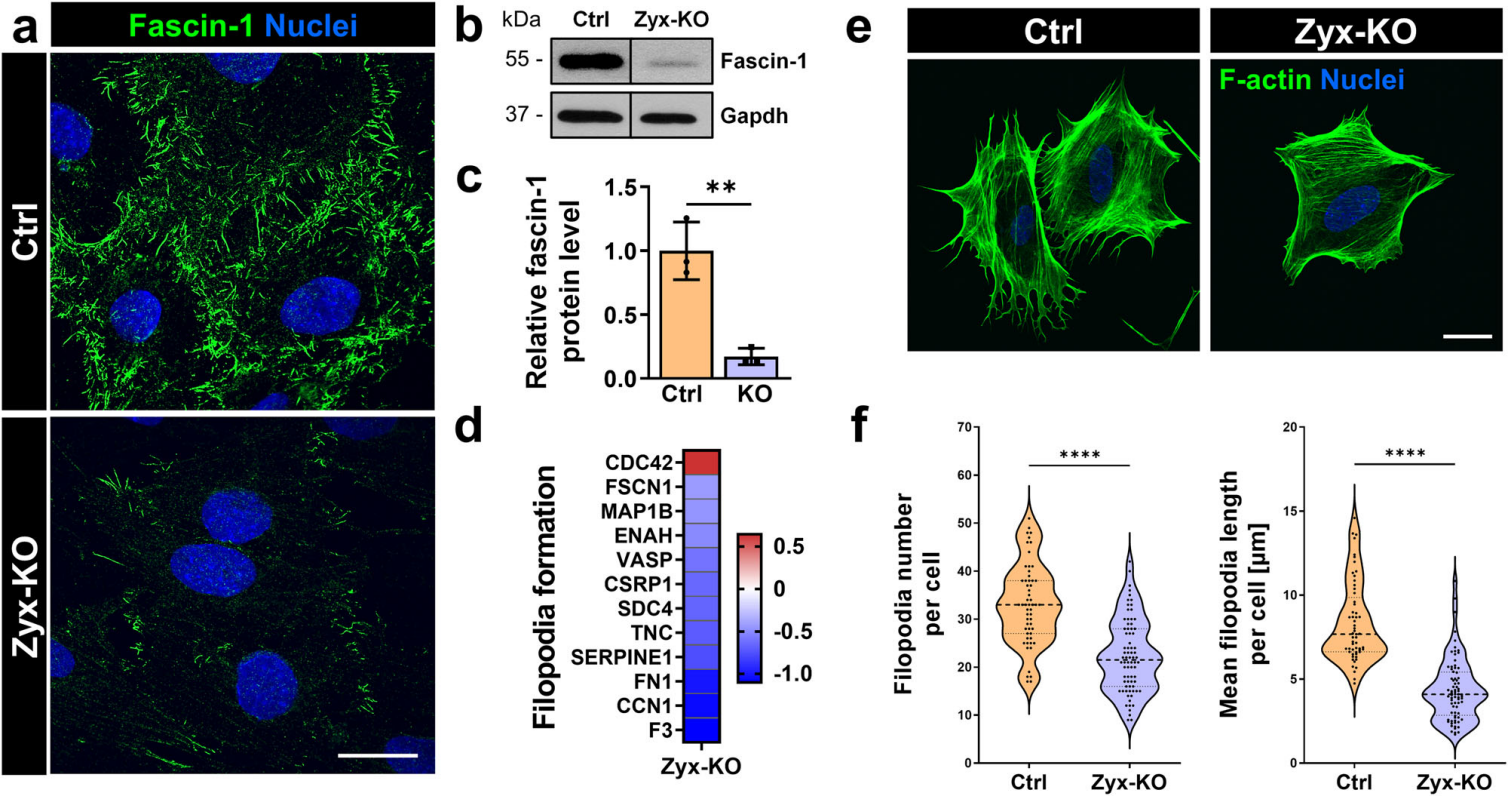
The loss of zyxin reduced the number and length of filopodia in cultured podocytes due to a down-regulation of fascin-1. **a** Immunofluorescence staining of Zyx-KO and control podocytes with an antibody against fascin-1 (shown in green). **b**, **c** Western blot with subsequent quantification of Zyx-KO podocytes showed a significant decrease of fascin-1 level compared to the control (Ctrl). Gapdh served as a loading control. Data are presented as means ± SD. **d** LC-MS/MS based relative protein level of different proteins involved in filopodia formation according to Ingenuity Pathway Analysis. Data are represented as log2 fold change, a *p* value < 0.05 was considered as significant (n = 3). Red: up-regulated; blue: down-regulated compared to controls. **e**, **f** Quantification of filopodia number and length. Each dot represents one individual cell (Ctrl: n = 57; Zyx-KO: n = 76). F-actin is shown in green. Nuclei stained with DAPI (shown in blue). Scale bars represent 25 μm. ** *p* < 0.01; **** *p* < 0.0001.

By immunostaining and Western blot analyses, we observed a significant decrease of the protein fascin-1 in Zyx-KO podocytes (Fig. 5a–c). Since fascin-1 is essential for the formation of filopodia in podocytes^46^, we speculated that the development of filopodia is altered due to the loss of zyxin. Analysis of the proteome data of Zyx-KO and Ctrl podocytes by using the Ingenuity Pathway Analysis^47^ tool predicted a reduction of the “filopodia formation” pathway. With the exception of Cdc42, many essential proteins involved in filopodia formation were significantly down-regulated in the Zyx-KO podocytes (Fig. 5d). Therefore, we quantified the number and length of the filopodia and found that the loss of zyxin leads to a reduction of the number (-35%) and mean length of the filopodia (-47%) in Zyx-KO podocytes compared to Ctrl (Fig. 5e, f).

### Zyxin is up-regulated in diabetic nephropathy (DN)

**Fig. 6:**
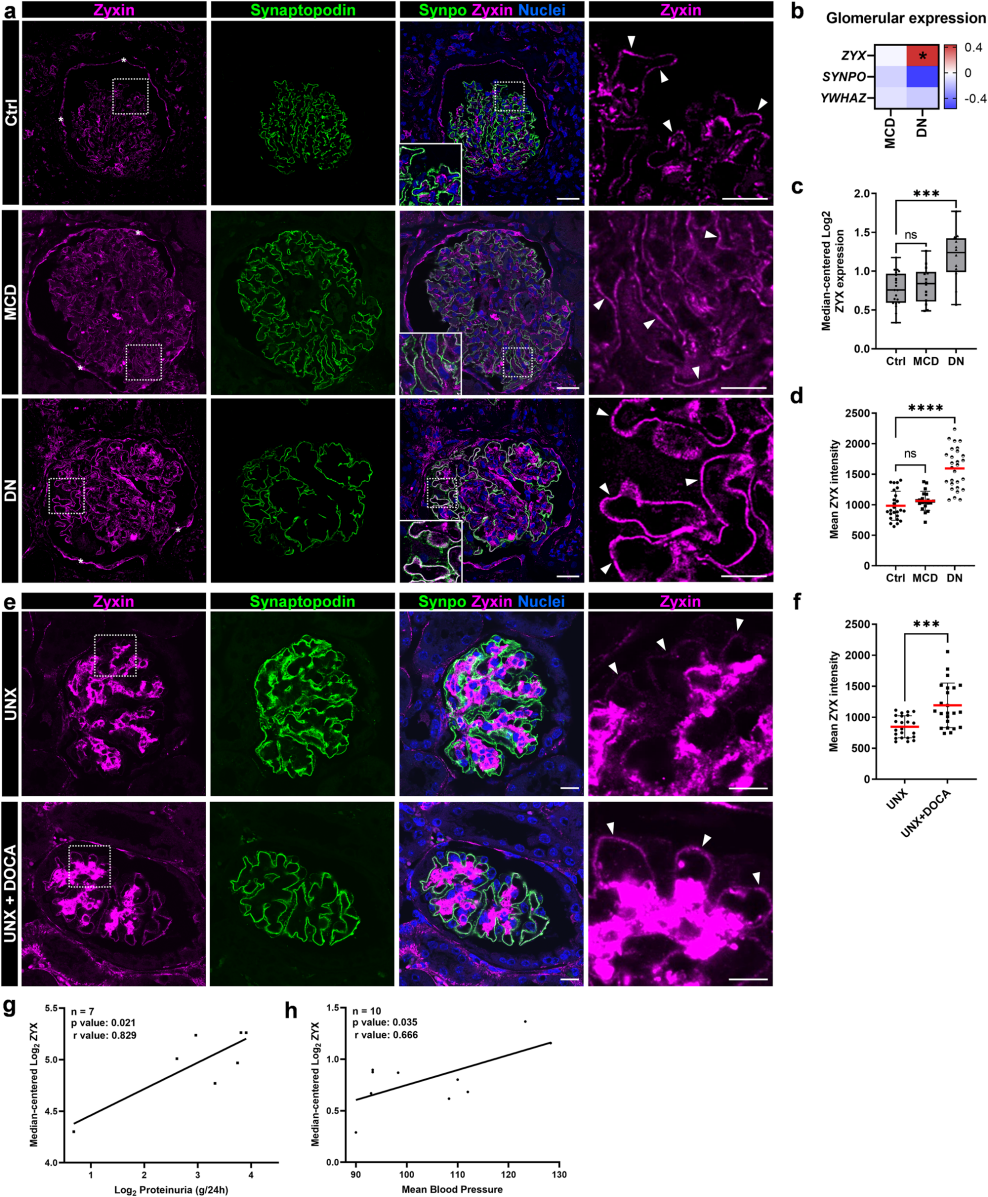
Zyxin is up-regulated in diabetic nephropathy and hypertensive mice. **a** The expression of zyxin (magenta) was strongly increased and colocalized (marked by white arrowheads) with synaptopodin (shown in green) in glomeruli of human patients suffering from DN (with clinically quantified hypertension) as compared to control tissue (Ctrl) and minimal change disease (MCD) imaged by LSM. Nuclei were stained with Hoechst (blue). Zyxin expression in PECs is marked by asterisks. Scale bars represent 25 µm and 10 µm (magnification), respectively. **b** mRNA expression level of microdissected glomeruli from renal biopsies of human patients suffering from diabetic nephropathy (DN; n = 14) and minimal change disease (MCD; n = 14) compared with healthy living donors (LD; n = 41). Data are represented as log fold changes, a q-value < 0.05 (*) was considered as significant. **c** Analysis of human glomerular disease data (Nephroseq database) further substantiated increased expression on mRNA levels of ZYX in DN (n = 12), but not in MCD (n = 14) patients compared to Ctrl (n = 21)^89^. **d** Mean ZYX fluorescence intensity in the foot processes of the podocytes in Ctrl, MCD and DN patients. Each dot represents the mean from one individual glomerulus (n ≥ 20). **e** Immunofluorescence staining showed a strong increase of zyxin (magenta; marked by arrowheads) and a co-localization with synaptopodin (green) in uninephrectomy (UNX) and desoxycorticosterone acetate (DOCA)-salt treated mice. Nuclei were stained with Hoechst (blue). Scale bars represent 100 µm and 50 µm (magnification). **f** Mean ZYX fluorescence intensity in the foot processes in UNX and UNX + DOCA mice (n = 23; analyzed glomeruli). **g** Zyxin expression correlates with proteinuria. The mRNA was isolated from laser-captured glomeruli from archived formalin-fixed, paraffin-embedded renal biopsies from focal segmental glomerulosclerosis (FSGS) patients and controls^90^. Data were taken from Nephroseq Research Edition (Ann Arbor, University of Michigan). **h** Zyxin expression correlates with blood pressure. Gene expression profiling of microdissected glomeruli samples was analyzed on an Affymetrix Human U133A GeneChip platform of 10 hypertension/nephrosclerosis samples^91^. Data were taken from www.nephroseq.org. * *p* < 0.05; *** *p* < 0.001; **** *p* < 0.0001.

To find out, whether zyxin is also regulated in human patients suffering from DN, we analyzed the mRNA expression of *ZYX* in micro-dissected glomeruli originated from renal biopsies of patients suffering from DN and compared them to mRNA levels of patients with Minimal Change Disease (MCD) and healthy living donors. We observed that patients with DN showed a significant increase of *ZYX* mRNA compared to the control tissue (log2 fold change: 0.434; Fig. 6b). In contrast, the zyxin expression in MCD patients did not change significantly (Fig. 6b). Analysis of human disease data confirmed the significant increase in the ZYX expression in DN, but not in MCD patients (Fig. 6c). Additionally, we observed a strong co-localization of zyxin with synaptopodin in the foot processes of the podocytes in glomeruli of patients suffering from DN associated with hypertension (Fig. 6a). Whereas zyxin in healthy living donors and in patient with MCD was only expressed slightly in podocytes, in DN patients, zyxin seemed to be expressed highly by podocytes (Fig. 6a). Furthermore, quantification of the mean ZYX fluorescence intensity in the foot processes of the podocytes also showed that zyxin expression increases podocyte-specifically in patients with DN (Fig. 6d).

In agreement with these data, we found an up-regulation of zyxin in the glomeruli of mice suffering from glomerular hypertension which was induced by uninephrectomy (UNX) and desoxycorticosterone acetate (DOCA)-salt treatment (Fig. 6e). We were able to confirm this by quantification of the mean Zyx intensity (Fig. 6f). Furthermore, data analysis has shown that zyxin expression is significantly correlated with proteinuria in FSGS, but not in MCD patients (Fig. 6g and Fig. S4) and blood pressure (Fig. 6h).

### *Zyx* KO mice show expansion of the mesangial compartment, a reduced slit membrane density and proteinuria

**Fig. 7:**
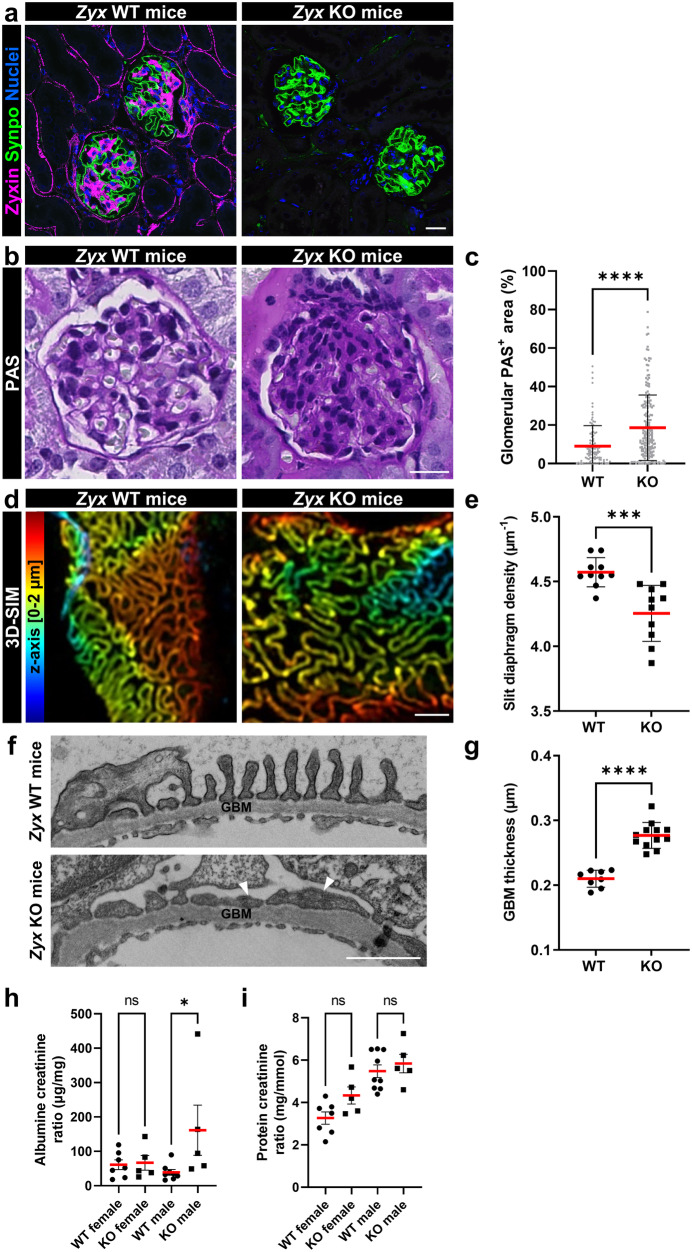
*Zyx* KO mice showed expansion of the mesangial compartment, reduced slit membrane density and proteinuria. **a** Immunofluorescence staining for zyxin (magenta) and the podocyte-specific marker synaptopodin (shown in green) were used to validate efficient deletion of zyxin in *Zyx* KO mice in contrast to *Zyx* wildtype (WT) mice. **b**, **c** Histologic evaluation of glomerular damage pattern employing Periodic acid–Schiff staining (PAS). At 6 months of age, expansion of the mesangial compartment was detectable. Assessment of glomerulosclerosis (glomerular PAS^+^ area) confirmed progressive glomerular damage (each dot represents one individual glomerulus (n ≥ 103); five WT and five KO animals (at 6 months of age) were analyzed). **d**, **e** 3D-SIM in *Zyx* KO animals identified a reduced filtration slit density (FSD) indicative for aberrant FP architecture. SIM data of 10 WT and 10 KO animals were quantified by PEMP (at 12 months of age). Kidney sections were stained for the slit diaphragm protein podocin. Z-axis scales of 3D-SIM were color coded as indicated. **f** Electron microscopy (EM) revealed a mild effacement of podocyte foot processes (arrowheads) at the glomerular basement membrane (GBM). **g** GBM thickness (in µm); dots indicate individual glomeruli (n ≥ 8). **h** Urinary albumin-creatinine ratio measurements indicated significantly increased levels of proteinuria in male *Zyx* KO mice at the age of 12 month after birth in comparison to male WT animals (each individual dot represents one experimental animal; n ≥ 5). **i** Protein creatinine ratio in mg/mmol (each individual dot represents one experimental animal; n ≥ 5). Scale bar represents 20 µm (**a**, **b**) or 1 µm (**d**, **f**). Data are presented as means ± SD (**c**, **e**, **g**) or means ± SEM (**h**, **i**). * *p* < 0.05; *** *p* < 0.001; **** *p* < 0.0001; ns, not significant.

After validation of the knockout of zyxin by immunostaining (Fig. 7a) we analyzed the glomeruli by PAS staining (Fig. 7b, c). At the age of 6 months, an expansion of the mesangial compartment was detectable (Fig. 7b). Assessment of glomerulosclerosis (glomerular PAS^+^ area) confirmed glomerular damage in *Zyx* KO mice (Fig. 7c). To determine more precisely the changes of the foot process morphology using super-resolution microscopy 3D-SIM, we applied the podocyte effacement measurement procedure on podocin-stained kidney sections (Fig. 7d), which was recently established by Siegerist et al. ^48^. In *Zyx* KO mice, we found a significant reduction of the slit diaphragm density compared to the control mice (4.25 µm^−1^ versus 4.57 µm^−1^) (Fig. 7e). By electron microscopy we were able to confirm the morphological glomerular changes. We observed a partial podocyte foot process effacement, pseudocysts, expansion of the mesangial cells and a thickening of the glomerular basement membrane in *Zyx* KO glomeruli (Fig. 7f, g and Fig. S5). Moreover, evaluation of albumin-creatinine ratios (ACR) showed a significant increase of proteinuria in male *Zyx* KO mice compared to the wildtype mice (ACR in µg/mg: WT: 39; KO: 161) (Fig. 7h). The protein-creatinine ratio was not changed significantly (Fig. 7i).

## Discussion

Podocyte damage and detachment caused by mechanical stress is a common issue in hypertension-induced glomerular disease^49,50^. However, it is unknown whether mechanical stress induces a dynamic change in the podocyte integrin adhesion complex or how such modulations affect the mechano-sensitive link with the GBM and the integrity of the glomerular filtration barrier. In this study, a comprehensive analysis of zyxin for signaling and cell adhesion highlighted the essential role of zyxin for podocyte stability and function.

We have found that zyxin is significantly up-regulated in podocytes after three days of mechanical stretch and relocalized from focal adhesions to actin filaments as well as to the actin-rich center (ARC), a characteristic structure in stretched podocytes^9^. Yoshigi and coworkers were the first to describe, that cyclic stretch or shear stress in vitro induces such a relocation of zyxin from focal adhesions to actin filaments in cultured fibroblasts^26^, whereas other focal adhesion proteins remained unchanged. In contrast, our zyxin knockout podocyte cell line showed a significant reduced size of vinculin-positive focal adhesions compared to control, whereas talin, a protein proposed to be a mechanosensor^51^, remained unchanged. A down-regulation of vinculin was also found in zyxin knockdown chondrocytes^52^. A translocation of zyxin into the nucleus, as it was observed by Gosh et al. in stretched vascular smooth muscle cells, was not found in podocytes^53^.

Since zyxin and vinculin can recruit Ena/VASP proteins by their EVH1 domain^32,54,55^, we studied the influence of zyxin on the localization and expression of VASP, a protein that plays a central role in cell adhesion, motility as well as in the regulation of integrin-extracellular matrix signaling pathways^56–58^. Especially the observation of different researcher that force can induce an accumulation of actin filaments at adhesion sites through a zyxin–VASP-dependent actin polymerization^59–61^ was highly interesting. Here, we show that zyxin is essential for the recruitment of VASP to actin filaments in podocytes as it was also described by Cheah et al., who found that VASP accumulated along force-bearing actin fibers in a zyxin-dependent manner^62^. We believe that the decrease of VASP and vinculin that were observed in Zyx-KO podocytes in contrast to other focal adhesion proteins such as α-actinin-4 and talin, led to an impaired cellular adhesion that we have seen during mechanical stretch. We found that 40% of the zyxin knockout podocytes detached during the three-day mechanical stress in contrast to the control cells. Since zyxin knockout podocytes also showed reduced extracellular matrix protein expression, such as fibronectin, periostin and nidogen, as well as a regulation of Rho GTPase-associated proteins (GEFs and GAPs), we hypothesized that zyxin might be a mechanosensor in podocytes. However, the number of ARC formations, a readout for mechanical stretch sensing of podocytes, was not changed indicating that zyxin is not a mechanosensor, but a mechanotransducer.

Small Rho GTPases also regulate the motility and spreading of cells^63–65^. Therefore, we looked whether the dynamics of zyxin knockout podocytes were affected by the knockout. We observed that the cell size as well as the motility were altered after the loss of zyxin, which is in accordance with data reported for vascular smooth muscle cells, fibroblasts and MDCK cells^56,61,66,67^.

In the past, we have observed that the actin-associated protein fascin-1, which is responsible for actin bundling, focal adhesion stability and filopodia formation by tightly crosslinking actin filaments into non contractile stress fiber^68–70^, is up-regulated by mechanical stretch^46^. Therefore, we studied the influence of zyxin expression on fascin-1. We found that the number and length of filopodia is significantly diminished after the loss of zyxin, suggesting that zyxin acts upstream of fascin-1. The down-regulation of fascin-1 might also reduce the stability of focal adhesion leading to the detachment of podocytes under mechanical stress.

The important role of zyxin in podocytes was underlined by in vivo results. We have seen that not only in diabetic nephropathy, a disease which is often associated with glomerular hypertension, but also in a classical glomerular hypertension model, the DOCA-salt treated mice, we have found a massive up-regulation of zyxin expression. We assume that zyxin is increasingly required in hypertensive glomerular injuries as a part of the mechano-adaptive response for podocytes to withstand mechanical stress. Furthermore, Z*yx* KO mice showed proteinuria and severe changes of podocyte foot process morphology, which were identified by super resolution microscopy and confirmed by electron microscopy.

Taken together, zyxin is an important protein that is critical for podocyte stability and function in vitro and in vivo, especially under mechanical stretch.

## Methods

### Cell culture

Conditionally immortalized podocytes (SVI; CLS Cell Line Service GmbH, Eppelheim, Germany) were handled as described previously^9^. Briefly, podocytes were maintained in RPMI-1640 medium (Sigma-Aldrich, St. Louis, MO, USA) supplemented with 10% fetal bovine serum (FBS; Boehringer Mannheim, Mannheim, Germany), 100 U/ml penicillin, and 0.1 mg/ml streptomycin (Thermo Fisher Scientific, Waltham, MA, USA). To propagate podocytes, we cultivated cells at 33 °C. To induce podocyte differentiation, we maintained podocytes at 38 °C for at least two weeks before applying mechanical stress.

### Mechanical stretch experiments

Mechanical stretch experiments were performed according to our previous study^9^. Differentiated mouse podocytes were seeded on flexible silicone membranes of a six well plate (Bioflex, Flexcell^®^ International Corporation, Burlington, NC, USA). The flexible silicone membranes were pre-coated with collagen IV to facilitate cell attachment. After three days, the six well plate was mounted on a manifold connected to a custom-built stretch apparatus (NIPOKA GmbH, Greifswald, Germany), which induced cyclic pressure variations resulting in upward and downward motion of the silicone membranes. Pressure amplitude was chosen to give a maximum upward deflection of the membrane center of 6 mm, being equal to an increase in membrane area by 11%, or 5% mean linear cell strain. Cycle frequency was adjusted to 0.5 Hz. This frequency was chosen because autoregulation of renal blood flow is capable of damping pressure fluctuations below 0.1 Hz, whereas faster pressure fluctuations ( > 0.1 Hz) are fully transmitted to glomerular capillaries^10,71^. Number of adherent cells was determined by semiautomatic-counting of stained podocytes with the StarDist ImageJ/Fiji Plugin^72^ from at least three biological replicates.

### Zyxin knockout podocytes

Cultured podocytes were transfected with CRISPR-Cas9 RNP complexes from Integrated DNA Technologies (Coralville, Iowa, USA) and Lipofectamine™ RNAiMAX as transfection reagent (Thermo Fisher Scientific) according to the manufacturer’s specifications. The following crRNAs were used: Mm.Cas9.ZYX.1.AD (ACTGGAGCAAACTTAGGTGC), Mm.Cas9.ZYX.1.AH (CGAGATCCCACCACCACCCC) or negative Control crRNA. After single cell clonal expansion zyxin knockout podocytes (Zyx-KO) were selected and verified by immunostaining, (q)RT-PCR, Western blot and DNA sequencing.

### Zyxin knockout mice (*Zyx* KO)

The *Zyx* KO mice were kindly provided by the group of M. Hecker^44^. The strain was originally designed by M. Beckerle^73^, but backcrossed more than 10-times onto the C57BL/6 J background. As wildtype control age-matched C57BL/6 J mice were used. Experiments were done with 6-month-old and 12-month-old male and female mice. All studies were carried out in strict accordance with regulations in Germany regarding the use of laboratory animals and were approved by the regional council in Karlsruhe.

### DOCA-salt induced hypertension

Induction of glomerular hypertension by uninephrectomy (UNX) and desoxycorticosterone acetate (DOCA) salt treatment has been described previously^74^.

### RNA extraction, cDNA synthesis and qRT-PCR

Samples were processed in Tri-Reagent (Sigma-Aldrich) according to manufacturer´s instructions. For cDNA synthesis, 1 µg of the isolated total RNA was transcribed using the QuantiTect Reverse Transcription Kit (Qiagen, Hilden, Germany). The quantitative real-time PCR (qRT-PCR) analysis was performed on a QuantStudio™ 5 Real-Time PCR System (Thermo Fisher Scientific) using the iTaq Universal SYBR Green Supermix (Bio-Rad) with *Gapdh* as reference gene. Relative quantifications of the mRNA levels were done by the efficiency corrected calculation model by Pfaffl^75^ and are shown with standard deviations (SD) or standard error of the mean (SEM) from at least three biological replicates. Used primers can be viewed in Supplementary Table 1.

### Western blot analysis

Protein lysates were separated using a 4-20% Mini-PROTEAN^®^ TGX™ Gel, (Bio-Rad Laboratories) and transferred to a nitrocellulose membrane using a Trans-Blot^®^ Turbo^™^ Transfer System (Bio-Rad Laboratories) for 10 min at 2 A. Membranes were immersed for 1 h in blocking buffer (10 mM Tris, 100 mM NaCl, 5% non-fat dry milk, 0.2% Tween-20, pH 7.5) and incubated overnight at 4°C in TBS-Tween (0.5%) with the following antibodies: anti-zyxin (Z4751, Sigma-Aldrich, St. Louis, MO, USA; immunoblot dilution 1:4000), anti-paxillin (610051, BD Biosciences; immunoblot dilution 1:25000), anti-talin (T3287, Sigma Aldrich; immunoblot dilution 1:10000), anti-vinculin (V9131, Sigma-Aldrich; immunoblot dilution 1:20000), anti-fibronectin (ab2413, Abcam; immunoblot dilution 1:2000), anti-fascin-1 (HPA005723, Sigma-Aldrich; immunoblot dilution 1:1000), anti-VASP (HPA005724, Sigma-Aldrich; immunoblot dilution 1:2000), anti-filamin A (SAB4500951, Sigma-Aldrich; immunoblot dilution 1:4000), anti-β-actin (sc-47778, Santa Cruz; immunoblot dilution 1:1000), anti-α-actinin-1 (A5044, Sigma-Aldrich; immunoblot dilution 1:2500), anti- α-actinin-4 (0042-05, immunoGlobe; immunoblot dilution 1:2000) and anti-Gapdh (10494-1-AP, Proteintech Group; immunoblot dilution 1:40000). Blots were incubated for 1 h at RT with HRP–conjugated secondary antibody anti-mouse (SA00001-1, Proteintech Group; immunoblot dilution 1:15000) or anti-rabbit (SA00001-2, Proteintech Group; immunoblot dilution 1:15000) and developed using Clarity™ Western ECL Blotting Substrate (Bio-Rad Laboratories) and finally exposed to X-ray films (Fujifilm Super RX, FUJIFILM, Tokyo, Japan). For the relative quantification, developed x-ray films were scanned and analyzed using the Fiji distribution of Image J (ImageJ J 1.51)^76,77^. Mean gray values of specific signals were determined and normalized to mean gray values of Gapdh signals.

### Liquid chromatography-mass spectrometry (LC-MS/MS)

Zyx-KO and control podocytes of three independent bioreplicates were harvested in 8 M urea/2 M thiourea and proteins extracted by five freeze-thaw cycles. Protein containing supernatant was collected by centrifugation (16,000 × *g*, 60 min, 20 ^o^C) and nucleic acid degraded enzymatically with universal nuclease 0.125 U/µg protein, (Pierce/Thermo, Rockford, IL, USA). Protein concentration was determined using a Bradford assay (Biorad). Four µg protein were reduced by 2.5 mM dithiothreitol for 1 h at 60°C and alkylated with 10 mM iodoacetic acid for 30 min at 37 °C in the dark. Samples were diluted with 20 mM ammoniumbicarbonate to 1 M urea/thiourea before digestion by trypsin (Promega, Walldorf, Germany) in a protein to enzyme ratio 25:1 overnight at 37°C. The reaction was stopped with acetic acid (1%) and the peptide mixtures were desalted on C-18 reverse phase material (ZipTip μ-C18 Millipore Corporation, Burlington, MA, USA). Peptide eluates were lyophilised and resuspended in 2% ACN in 0.1% acetic acid. Peptides were separated by LC (Ultimate 3000, Thermo Electron, Bremen, Germany) before data-independent acquisition of MS data on an Exploris 480 mass spectrometer (Thermo Electron). MS data were analysed via the DirectDIA algorithm implemented in Spectronaut (v15, Biognosys, Zurich, Switzerland) using an Uniprot database (version 2021_2) limited to *Mus musculus* (n = 17,063). Carbamidomethylation at cysteine was set as static modification, oxidation at methionine and protein N-terminal acetylation were defined as variable modifications, and up to two missed cleavages were allowed. Proteins were only considered for further analyses, if two or more unique+razor peptides were identified and quantified per protein. Data were median normalized on ion level before statistical analysis was carried out on peptide level after exclusion of peptides with oxidized methionine using the algorithm ROPECA^78^. Binary differences were identified by application of a reproducibility-optimized test statistic (using the ROTS package). Only proteins that showed different abundance (*p* value < 0.05) were used for further considerations. Detailed description of data acquisition and search parameters are provided in Supplementary tables 2 and 3. Furthermore, data were analysed by Gene Set Enrichment Analysis (GSEA) and the use of QIAGEN IPA (QIAGEN Inc.). For the analysis of the differentially abundant proteins R scripts including the libraries EnhancedVolcano and ComplexHeatmap were used for the visualization. The heatmaps include on the Y-axis a dendrogram of the hierarchical clustering after Z-score normalization of the protein abundance values in the different samples. The heatmaps are used to represent the abundance of proteins as ratio from treatment versus control of specific biological pathways from the GeneOntology (GO). To detect the enriched and overrepresented pathways and their protein protein interactions the cnetplots and dotplots from the GO biological processes were created using the R library clusterProfiler^79^.

### Immunocytochemistry

Cells were fixed using 2% paraformaldehyde (PFA) in phosphate-buffered saline (PBS) for 10 min at room temperature (RT), washed and subjected to blocking solution (PBS, 2% fetal bovine serum, 2% bovine serum fraction V, 0.2% fish gelatin) for 1 h at RT. Primary antibodies were incubated for 60 min at RT. The following antibodies were used: anti-zyxin (Z4751, Sigma-Aldrich; IF dilution 1:200), anti-paxillin (610051, BD Biosciences; IF dilution 1:200), anti-talin (T3287, Sigma-Aldrich; IF dilution 1:100), anti-vinculin (V9131, Sigma-Aldrich; IF dilution 1:400), anti-fibronectin (ab2413, Abcam; IF dilution 1:250), anti-fascin-1 (HPA005723, Sigma-Aldrich; IF dilution 1:100) and anti-VASP (HPA005724, Sigma-Aldrich; IF dilution 1:200). Actin was stained using Alexa Fluor^®^ 488 phalloidin (Thermo Fisher Scientific; dilution 1:100). After a washing step with PBS (3 × 3 min) cells were incubated with secondary antibodies for 45 min at RT. Bound antibodies were visualized with Cy2- or Cy3-conjugated secondary antibodies (Jackson ImmunoResearch Laboratories, West Grove, USA; IF dilution 1:300). For staining of nuclei DAPI (Sigma-Aldrich, 1 µg/ml) was used for 5 min. All samples were mounted in Mowiol (Carl Roth, Karlsruhe, Germany) and used for laser scanning microscopy (LSM). Pictures for focal adhesion analysis were taken randomly on the microscope slides.

### Histology

For paraffin sections, samples were dehydrated and embedded in paraffin by standard procedures. Paraffin sections (2 µm) were cut on a Leica SM 2000R (Leica Microsystems, Wetzlar, Germany). After rehydration, sections were unmasked in citrate buffer (0.1 M, pH 6.0) by heating for 5 min in a pressure cooker. The nuclei were stained with 1 mg/100 ml Hoechst 33342 (Sigma-Aldrich) for 30 min. For immunofluorescence double-staining, samples were incubated with an antibody against zyxin (HPA004835 for human and HPA073497 for mice FFPE material; both from Sigma-Aldrich; IF dilution 1:100) and synaptopodin (61094, Progen Biotechnik GmbH, Heidelberg, Germany; IF dilution 1:100) overnight. Samples were washed with 1x PBS for 3 × 5 min and incubated with Cy2- and Cy3-conjugated secondary antibodies (Jackson ImmunoResearch Laboratories; IF dilution 1:300) for 1 h. After additional washing, the samples were mounted in Mowiol (Carl Roth) for fluorescence microscopy. PAS stainings were performed by standard procedures. For transmission electron microscopy, kidneys were embedded in EPON 812 (SERVA, Heidelberg, Germany). Ultrathin sections were cut and contrasted with 5% uranyl acetate and lead citrate. All grids were examined with a LIBRA^®^ 120 transmission electron microscope (Carl Zeiss Microscopy, Jena, Germany). Scanning electron microscopy was performed according to Artelt et al. ^80^

### Histology on human kidney biopsies

Kidney biopsies were received from the Department of Nephropathology, Institute of Pathology, University Hospital Erlangen, Germany. The use of remnant kidney biopsy material was approved by the Ethics Committee of the Friedrich-Alexander-University of Erlangen-Nürnberg, waiving the need for retrospective consent for the use of archived rest material (No. 22-150-D). Sample fixation (4% PBS-buffered formalin pH 7.6) and preparation of the control, minimal change disease (MCD) and diabetic nephropathy (DN) group were identical. All MCD patients (n = 3) had proteinuria (1,7–3,2 g/d) and effacement of the podocyte foot processes (examined by electron microscopy).

### Microarrays on human kidney biopsies

Human renal biopsy specimens and Affymetrix microarray expression data were procured within the framework of the European Renal cDNA Bank - Kröner-Fresenius Biopsy Bank^81,82^. Biopsies were obtained from patients after informed consent and with approval of the local ethics committees. Following renal biopsy, the tissue was transferred to RNase inhibitor and microdissected into glomeruli and tubulointerstitium. Total RNA was isolated from microdissected glomeruli, reverse transcribed, and linearly amplified according to a protocol previously reported^83^.

Previously generated microarray data from microdissected human glomeruli sourced from individuals with kidney disease and healthy donors were used (GEO accession numbers: GSE32591, GSE37463, GSE47185, GSE99340). Pre-transplantation kidney biopsies from living donors were used as control. CEL file normalization was performed with the Robust Multichip Average method using RMAExpress (version 1.20) and the human Entrez‐Gene custom CDF annotation from Brain Array (version 25). The log-transformed dataset was corrected for batch effect using ComBat from the GenePattern pipeline. To identify differentially expressed genes, the SAM (Significance Analysis of Microarrays) method was applied using SAM function in Multiple Experiment Viewer (TiGR MeV, Version 4.9)^84^. A q-value below 5% was considered to be statistically significant. Analysis included gene expression profiles from patients with diabetic nephropathy (n = 14), minimal change disease (n = 14) as well as controls (living donors, n = 41).

### Image analysis

For quantification of focal adhesions size, we developed the custom software “Focal Contact Segmentation and Analysis Tool”. The focal contacts (FC) are segmented by a gradient-based local-threshold method. The specificity of this segmentation is further increased by relating the peripheral region of each FC to its center intensity. Slightly connected FCs are separated. Finally, specific shape parameters and the fluorescence activities of each FCs have been computed. Additionally, the cells are segmented by a semi-automatic technique and therefore the exact position of each FC within the cell is known^46,85^.

### Podocyte foot process effacement measurement procedure

The evaluation of the filtration slit density was performed using a recently established super-resolution microscopy-based methodology (structured illumination microscopy, SIM; N-SIM [Nikon] with a 100× silicone objective) termed as podocyte exact morphology measurement procedure (PEMP)^48^. The three-dimensional (3D) SIM (z-stack) images of slit diaphragms were colorized according to their position on the z axis. Filtration slit density values of six glomeruli in five mice per group were quantified. The slit diaphragm density was stated as length of the slit diaphragm per glomerular capillary area in *µ*m^−1^.

### Confocal laser scanning microscopy

Images were captured with an Olympus FV3000 confocal microscope (Olympus, Tokyo, Japan) with 20x/40x/60x oil immersion objectives and Olympus FV3000 CellSense software.

### Deep-learning enhanced cell migration assay

Cells were cultured in flat-bottom 24 well plates. Medium was exchanged to phenol-free medium containing 100 ng/ml HOECHST 33342 and image sequences (a single frame every 20–30 min were acquired in the Acquifer Imaging Machine (Acquifer GmbH, Heidelberg, Germany)). Image sequences were imported to FIJI^76^ and cell nuclei detected using the StarDist extension^72^ and an elsewhere published custom-trained StarDist network^86^. Cell tracks were merged and random cell velocity was quantified with the TrackMate plugin^87,88^.

### Statistics and reproducibility

The GraphPad Prism 9 software was used for statistical analysis of experimental data and preparation of graphs. Scatter plots indicate individual units used for statistical testing (samples, cells or replicates), as specified in the respective figure legends. Data are given as means ± SD or ±SEM, analyzed by unpaired *t* test with repeated measurements (n). For multiple groups statistical analyses were done by ANOVA followed by a Benjamini-Hochberg post-hoc test. Statistical significance was defined as p < 0.05 and significance levels are indicated as *p < 0.05, **p < 0.01, ***p < 0.001, ****p < 0.0001 or non-significant (ns). To verify the results, we conducted the experiments with n ≥ 3 to confirm their reproducibility. The exact number of independent experiments and analyzed units are stated in the figure legends.

### Reporting summary

Further information on research design is available in the Nature Portfolio Reporting Summary linked to this article.

### Supplementary information

Supplementary information

Description of Additional Supplementary Files

Supplementary Data 1

Supplementary Data 2

Reporting Summary

## Data Availability

All data supporting the findings of this study are available within the paper and its Supplementary Information. The mass spectrometry proteomics data are available in supplementary data files (Supplementary Data 1) and have been deposited to the ProteomeXchange Consortium via the PRIDE partner repository with the dataset identifier PXD050861. All uncropped blots and raw data are available within the article and its supplementary data files (Fig. S6 and Supplementary Data 2). All other relevant data and materials are available from the corresponding author on reasonable request.
