# Supplementary material for: Zyxin is important for the stability and function of podocytes, especially during mechanical stretch
Source: Commun Biol. 2024 Apr 11;7:446. doi: 10.1038/s42003-024-06125-5 (PMC11009394; doi:10.1038/s42003-024-06125-5)
Supplement: Supplementary file 1 — Supplementary information [file 42003_2024_6125_MOESM1_ESM.pdf]

Zyxin is important for the stability and function of podocytes, especially during mechanical stretch

Felix Kliewe[1*], Florian Siegerist[1], Elke Hammer[2], Jaafar Al-Hasani[3], Theodor Rolf Jakob Amling[1], Jonas Zeno Eddy Hollemann[1], Maximilian Schindler[1], Vedran Drenic[4], Stefan Simm[5], Kerstin Amann[6], Christoph Daniel[6], Maja Lindenmeyer[7,8], Markus Hecker[3], Uwe Völker[2], Nicole Endlich[1,4]

[1]Department of Anatomy and Cell Biology, University Medicine Greifswald, Greifswald, Germany
[2]Interfaculty Institute for Genetics and Functional Genomics, University Medicine Greifswald, Greifswald, Germany
[3]Department of Cardiovascular Physiology, Heidelberg University, Heidelberg, Germany.
[4]NIPOKA GmbH, Center of High-End Imaging, Greifswald, Germany.
[5]Institute of Bioinformatics, University Medicine Greifswald, Greifswald, Germany.
[6]Department of Nephropathology; Friedrich-Alexander University (FAU) Erlangen-Nuremberg, Erlangen, Germany
[7]III. Department of Medicine, University Medical Center Hamburg-Eppendorf, Hamburg, Germany.
[8]Hamburg Center for Kidney Health (HCKH), University Medical Center Hamburg-Eppendorf, Hamburg, Germany

[*] Corresponding author:

Dr. Felix Kliewe

Institut für Anatomie und Zellbiologie, Universitätsmedizin Greifswald

Friedrich-Loeffler-Str. 23c, 17489 Greifswald, Germany

Email: felix.kliewe@uni-greifswald.de

Supplementary information

Supplementary Figures

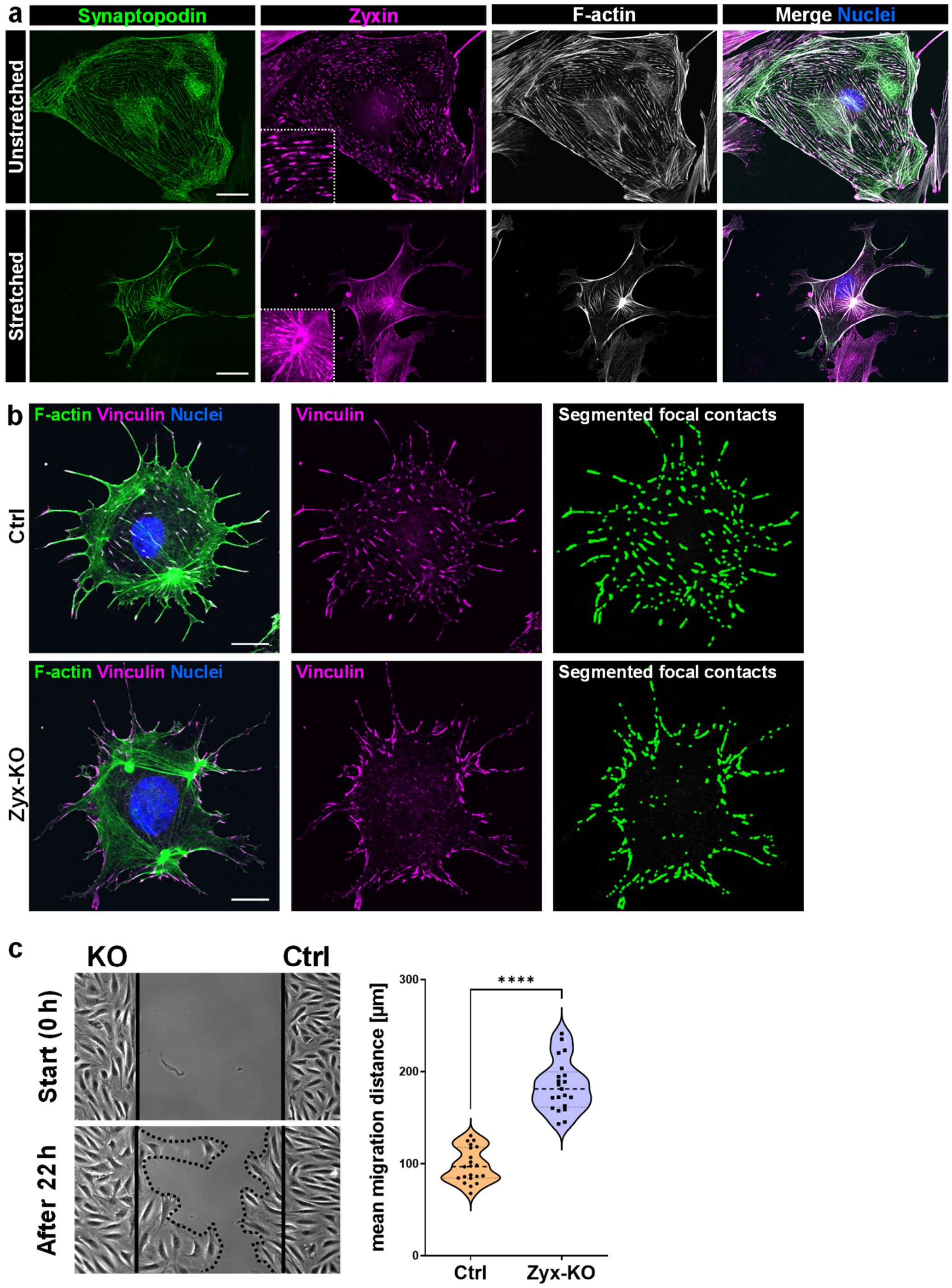

Fig. S1: Additional information for figures 1, 2 and 4.

(a) Mechanically stretched primary podocytes showed an increased zyxin (magenta) localization from focal contacts to actin filaments (white). Primary podocytes showed no zyxin accumulation in the nucleus after mechanical stretch. Synaptopodin (green) was used as a podocyte control. The scale bar represents 25 µm. (b) Immunostaining of vinculin (magenta) and F-actin (green) in Ctrl and Zyx-KO mechanically stretched immortalized podocytes. For quantification vinculin-positive signals were segmented and quantified (Fig. 2h). The scale bar represents 100 µm. (c) Migration assays were performed to study the migration of Zyx KO podocytes. For this, Zyx KO and Ctrl podocytes were cultured in a migration chamber as confluent layers which were separated into two distinct areas by a removable plastic partition in the middle of the chamber. After removing the partition, the dynamics of podocytes were followed over 22 hours by light microscopy. Images show different time points (0 and 22 hours). The distance between the migrating areas is marked by black (dashed) lines and revealed that Zyx KO podocytes migrated significantly faster into the gap than the control podocytes. Data are presented the mean values of individual field of views (n=21; **** *p*<0.0001).

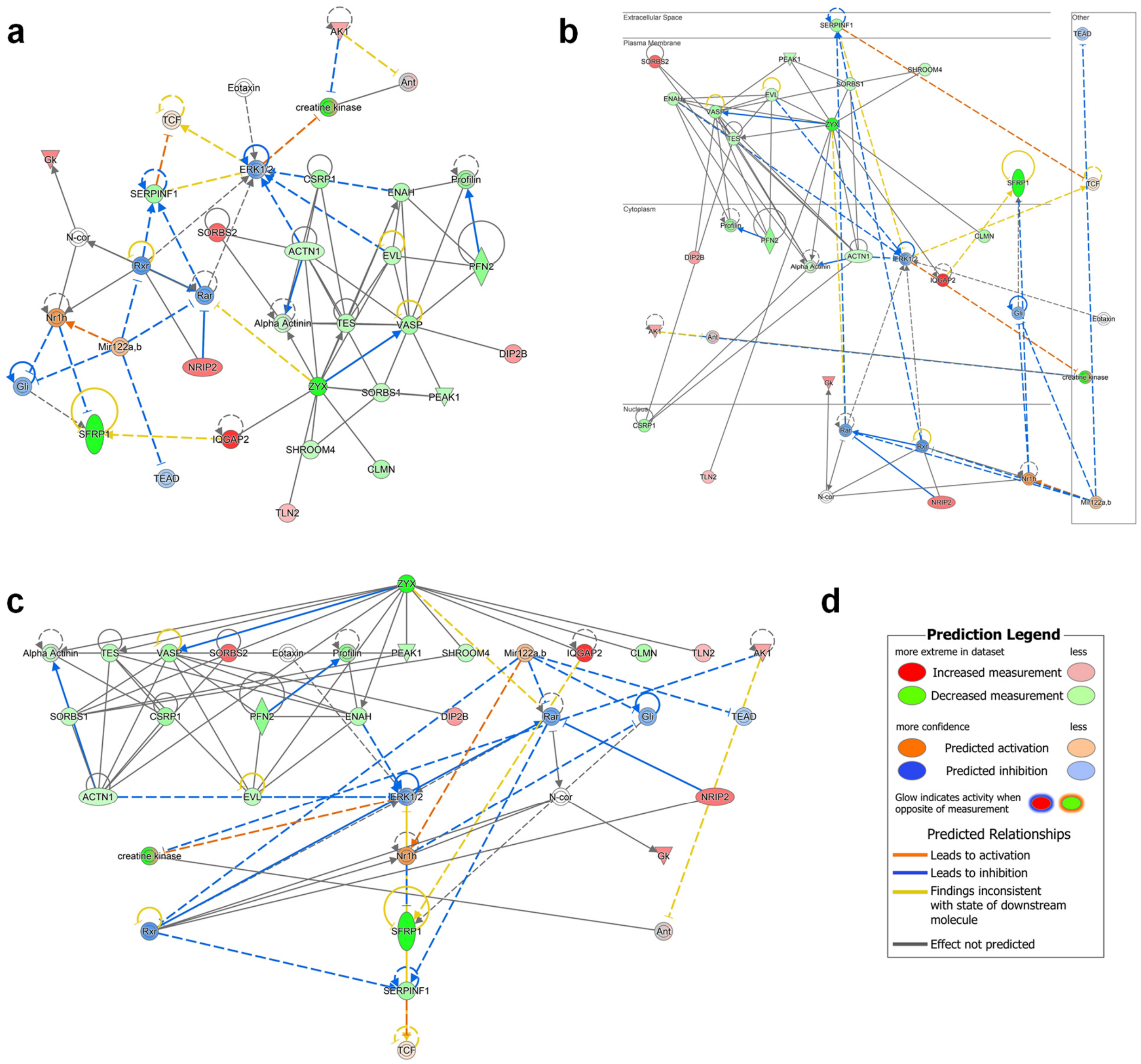

Fig. S2: Ingenuity Pathway Analysis (IPA).

IPA was used to identify zyxin containing protein-protein interaction networks. (a) Organic representation of the interaction network. The organic layout style is based on the force-directed layout paradigm. (b) Subcellular illustration places the nodes into a simplified view of subcellular compartments (c) Hierarchical representation of the interaction network highlights the main flow within a directed graph. (d) Prediction Legend: Red: up-regulated; green: down-regulated in Zyx-KO podocytes compared to controls (measured and quantified by LC-MS/MS). Orange: predicted up-regulation; blue: predicted down-regulation.

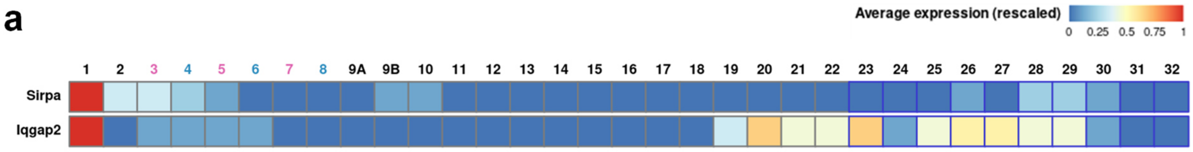

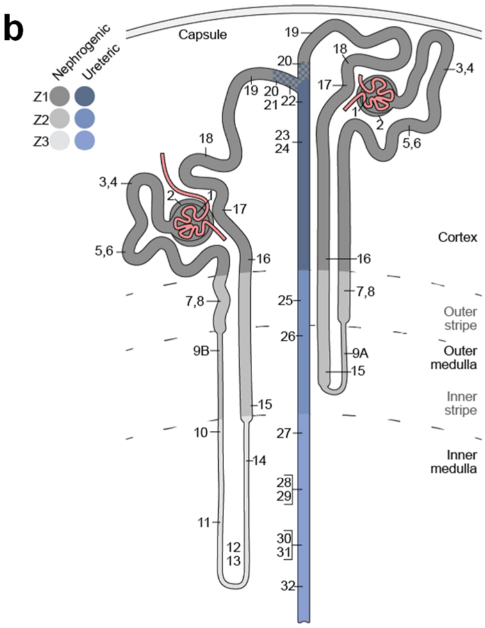

ID Ontology

1 podocytes (visceral epithelium)
2 parietal epithelium
3 segment 1 of proximal tubule - female
4 segment 1 of proximal tubule - male
5 segment 2 of proximal tubule - female
6 segment 2 of proximal tubule - male
7 segment 3 of proximal tubule - female
8 segment 3 of proximal tubule - male
9A LOH thin descending limb of inner stripe of outer medulla of cortical nephron
9B LOH thin descending limb of inner stripe of outer medulla of juxtamedullary nephron
10 upper LOH thin descending limb of inner medulla of juxtamedullary nephron
11 lower LOH thin descending limb of inner medulla of juxtamedullary nephron
12 lower LOH thin limb of inner medulla of juxtamedullary nephron
13 lower LOH thin limb of inner medulla of juxtamedullary nephron
14 upper LOH thin ascending limb of inner medulla of juxtamedullary nephron
15 distal straight tubule of inner stripe of outer medulla (syn: thick ascending limb of LOH)
16 distal straight tubule of outer stripe of outer medulla and cortex
17 macula densa
18 distal convoluted tubule
19 nephron connecting tubule
20 principal-like cell of nephron connecting tubule
21 intercalated type non-A non-B cell of nephron connecting tubule
22 intercalated type A cell of nephron connecting tubule and cortical collecting duct
23 principal-like cell of cortical collecting duct
24 intercalated type B cell of cortical collecting duct
25 intercalated type A cell of outer medullary collecting duct
26 principal cell of outer medullary collecting duct
27 intercalated type A cell of inner medullary collecting duct
28 principal cell of inner medullary collecting duct type 1
29 principal cell of inner medullary collecting duct type 2
30 principal-like cell of deep inner medullary collecting duct type 1
31 cell of deep inner medullary collecting duct type 2
32 deep medullary epithelium of pelvis

Fig. S3: Kidney expression pattern of Sirpa and Iqgap2.
(a) Database analysis using the Kidney Cell Explorer by Ransick et al. based on a single-cell RNA sequencing data set of murine kidneys showed the expression pattern for all kidney cell fractions [PMID: 31689386]. Colour code: Red means a high average expression; Blue: low expression. (b) Legend of the numerical labelling of Fig. S3a. Data and illustrations are from KidneyCellExplorer (https://cello.shinyapps.io/kidneycellexplorer/; Ransick et al., 2019).

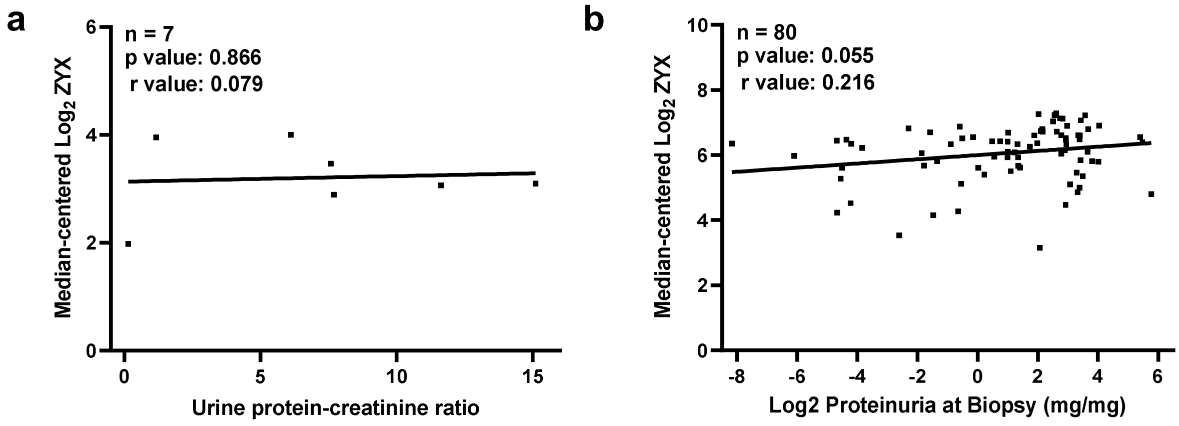

Fig. S4: Correlation of zyxin expression with proteinuria (Minimal Change Disease Samples).
Zyxin expression is not correlated with urine protein-creatine ratio (a) and proteinuria (b) in different MCD patient cohorts. (a) The data were analyzed using an Affymetrix Human Gene 2.1 ST Array [PMID: 26150607]. (b) Data were analyzed by RNA_Seq [PMID: 36442540]. Both data sets were taken from Nephroseq Research Edition (Ann Arbor, University of Michigan).

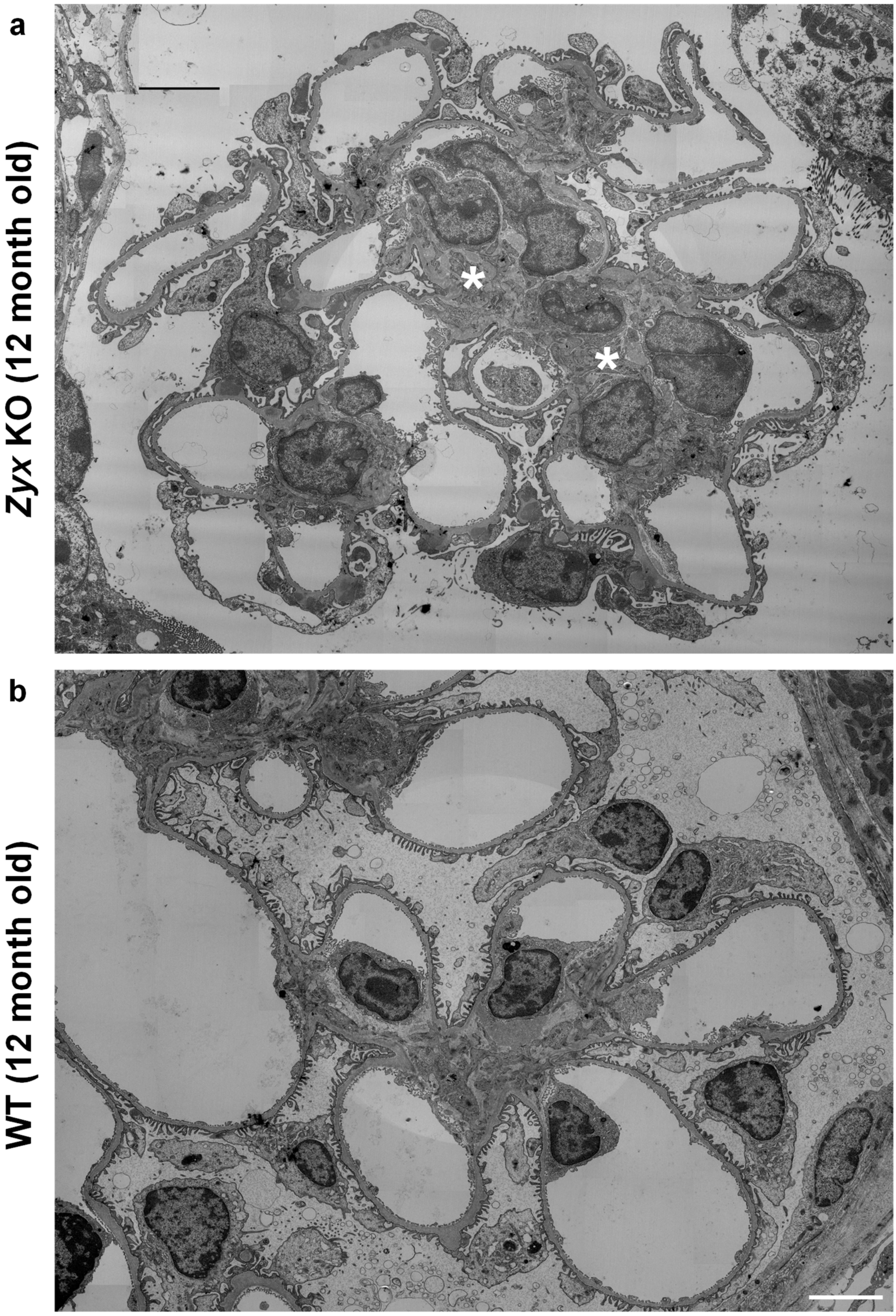

Fig. S5: Electron microscopy analysis of zyxin knockout mice.

Electron microscopy images from (a) male *Zyx* KO mouse at the age of 12 month after birth in comparison to (b) 12-month-old male wildtype (WT) mouse revealed partial podocyte foot process effacement, pseudocysts, expansion of the mesangial cells (marked by asterisks) and a thickening of the glomerular basement membrane in *Zyx* KO glomeruli. Scale bar represents 5 µm.

Fig. 2b

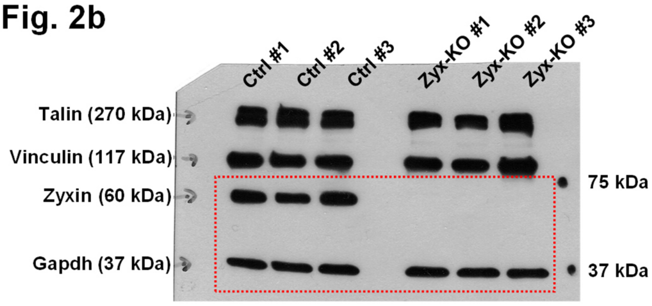

Fig. 3a

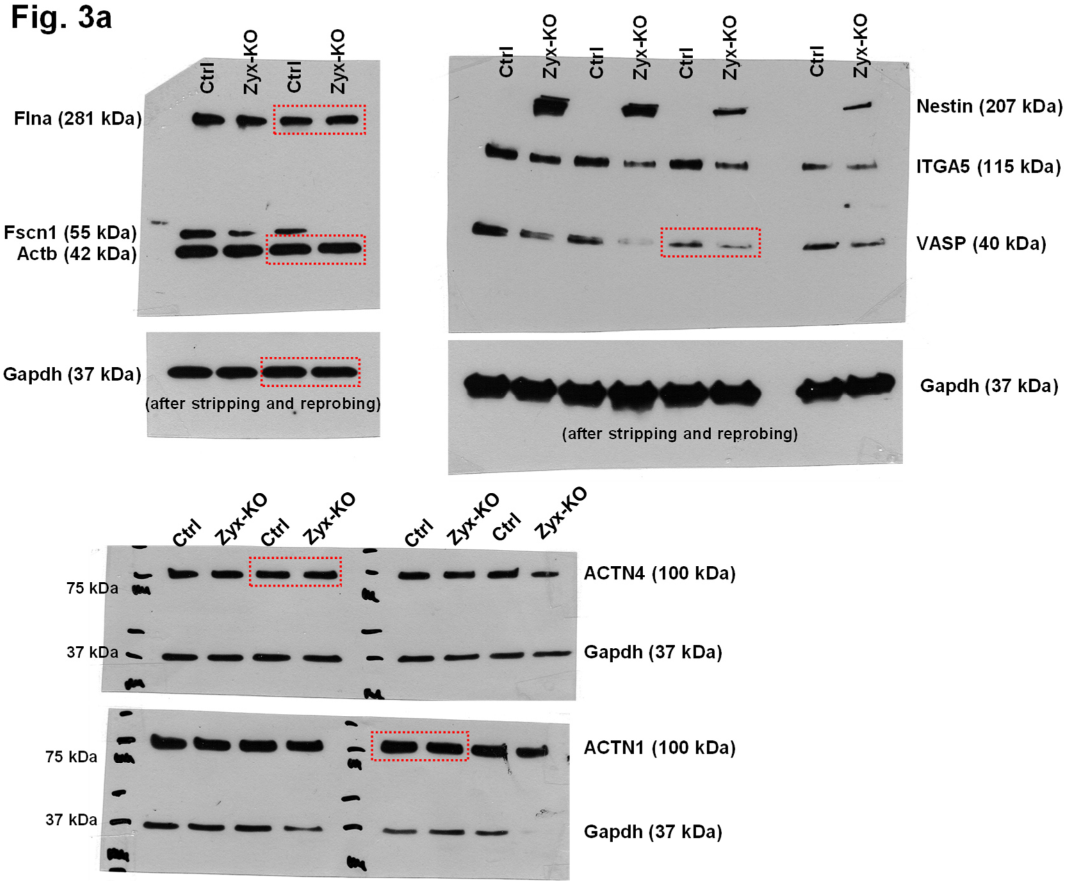

Fig. 5b

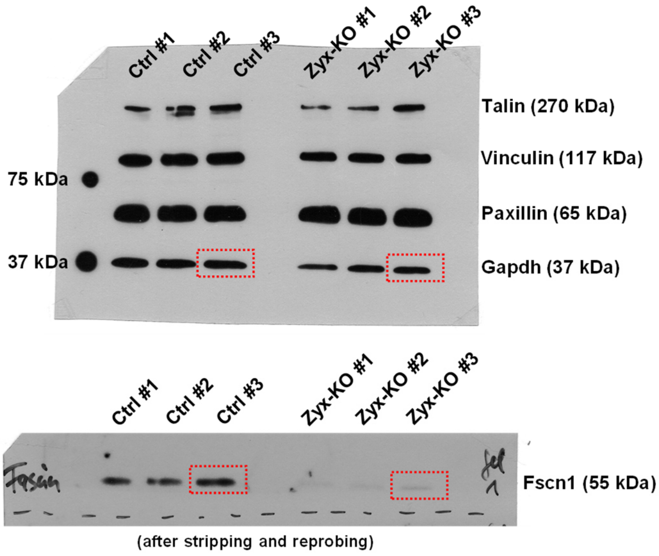

Fig. S6: Uncropped Western blots of the figures presented in Main Figures.

Supplementary Tables
Supplementary Table 1:

Used qRT-PCR Primer

Detected gene	Sequence / Identity
Gapdh	F: GGGTTCCTATAAATACGGACTGC<br>R: ATGAAGGGGTCGTTGATGGC
Zyx	F: GCTGCAGGGACAGCAGTTCT<br>R: CTGTGGGTGGTAGGCTTTGC
Tln1	F: AAGGCTCTCTGTGGCTTCAC<br>R: CTGACAGGCCATCTGAATTG
Vcl	F: AGAACTCACTCCCCAGGTCAT<br>R: CCACTGGTTCTTCATGGTCTC
Pxn	F: AGCCTCTTGGATGAACTGGA<br>R: ATCTGACAGTGACGCCATGA
Sirpa	F: CTCGTAGTCCTGCTGATGGC<br>R: ATCTGGGTTATTTCCCTGGCG
Iqgap2	F: GCCCAGAGGTGGGATCGG<br>R: GTTCTGTCGCCTCCTCTCATC
Nid2	F: CGCTATCGCTGCTGGTGATG<br>R: GTGGAGATGATGCCGTTGGT
Thbs1	F: AATCATGGCTGACTCGGGAC<br>R: GCGCTGGTTATGATTGGCAG
Postn	F: AAGGCGAAACGGTGACAGAA<br>R: CCTCCTGTGGAAATCCTGGT

Supplementary Table 2:

LC-MS/MS parameter (data independent mode; quantitative data)

Data independent analyses	
reversed phase liquid chromatography	**Ultimate 3000 RSLC (Thermo Scientific)**
Trap column	75 µm inner diameter, packed with 3 µm C18 particles (Acclaim PepMap100, Thermo Scientific)
Analytical column	75 µm inner diameter, packed with 2.6 µm C18 particles (Accucore, 25 cm, Thermo Scientific)
Flow rate	300 nl/min
column oven temperature	40°C
buffer system	binary buffer system consisting of 0.1% acetic acid in HPLC-grade water (buffer A) and 100% ACN in 0.1% acetic acid (buffer B)
gradient	gradient of buffer B: 2min 2% to 5 %, 8min 7%, 60min 7% to 25%, 5min 25 to 40%, 2 min 40% to 90%, 6 min 90%, 2 min 90% to 2%, 10 min 2%
Mass spectrometer	Exploris 480
operation mode	data-independent
electrospray	Nanospray Flex Ion Source
Full MS	
MS scan resolution	120,000
Normalized AGC target	300 %
maximum ion injection time for the MS scan	60 ms
Scan range	350 to 1200 *m/z*
Spectra data type	profile
dd-MS2	
Resolution	30,000
Normalized MS/MS AGC target	3000
maximum ion injection time for the MS/MS scans	auto
Spectra data type	profile
selection for MS/MS	1
isolation window	65 windows *m/z* 13 (overlap *m/z* 2)
Fixed first mass	200
dissociation mode	higher energy collisional dissociation (HCD)
normalized collision energy	fixed, 30
dissociation mode	HCD

Supplementary Table 3:

Spectronaut parameters for peptide/protein identification and intensity extraction

Spectronaut 15.5.211111.50606	
Computer Name: AGVOE-SPECTRONA	
User Domain Name: AGVOE-SPECTRONA	
User Name: spectronaut	
Analysis Mode: UI	
Analysis Type: directDIA	
Analysis Date: 30-December-2021 10:51:00 UTC+1	
Database: Uniprot-reviewed; Mus musculus; v. 21-02-2221	

Settings Used: C_FunGene_directDIA_sparse_no_imputing	
DIA Analysis\Calibration	
MZ Extraction Strategy:	Maximum Intensity
Allow source specific iRT Calibration:	True
Precision iRT:	True
Exclude De-amidated Peptides:	True
iRT <-> RT Regression Type:	Local (Non-Linear) Regression
MS1 Mass Tolerance Strategy:	System Default
MS2 Mass Tolerance Strategy:	System Default
DIA Analysis\Identification	
Precursor Qvalue Cutoff:	0.001
Precursor PEP Cutoff:	0.2
Protein Qvalue Cutoff (Experiment):	0.01
Protein Qvalue Cutoff (Run):	0.05
Protein PEP Cutoff:	0.75
Single Hit Definition:	By Stripped Sequence
Exclude Single Hit Proteins:	False
Exclude Duplicate Assays:	True
Exclude Predicted Fragment Scores:	False
Generate Decoys:	True
Decoy Generation Method:	Mutated
Preferred Fragment Source:	NN Predicted Fragments
Decoy Limit Strategy:	Dynamic
Library Size Fraction:	0.1
Pvalue Estimator:	Kernel Density Estimator
DIA Analysis\Pipeline Mode	
Generate SNE File:	True
Store Ion traces in SNE:	False
Post Analysis Reports:	
CV Density Line Chart:	True
CVs Below X Bar Chart:	True
Data Completeness Bar Chart:	True
Run Identifications Bar Chart:	True
Scoring Histograms:	True

Report Schema:	C_FunGene_complex (Normal)
Reporting Unit:	Across Experiment
DIA Analysis\Post Analysis	
Differential Abundance Testing:	NA
Group-Wise Testing Correction:	False
Differential Abundance Grouping:	Major Group (Quantification Settings)
Smallest Quantitative Unit:	Precursor Ion (Quantification Settings)
Use All MS-Level Quantities:	False
Calculate Explained TIC:	None
Calculate Sample Correlation Matrix:	True
Hierarchical Clustering:	True
Distance Metric:	Manhattan Distance
Linkage Strategy:	Ward's Method
Order Runs by Clustering:	True
Z-score Transformation:	False
DIA Analysis\Protein Inference	
Protein Inference Workflow:	Automatic
Inference Algorithm:	IDPicker
DIA Analysis\PTM Workflow	
PTM Localization:	False
DIA Analysis\Quantification	
Precursor Filtering:	Identified (Qvalue)
Imputation Strategy:	Use Background Signal
Proteotypicity Filter:	None
Protein LFQ Method:	MaxLFQ
Quantity MS Level:	MS2
Quantity Type:	Area
Cross-Run Normalization:	True
Normalization Filter Type:	None
Normalization Strategy:	Local Normalization
Row Selection:	Identified in at least 1 Run (Sparse)
Interference Correction:	True
Only Identified Peptides:	True
Exclude All Multi-Channel Interferences:	True
MS1 Min:	2
MS2 Min:	3
Major (Protein) Grouping:	by Protein Group Id
Minor (Peptide) Grouping:	by Stripped Sequence
Major Group Quantity:	Mean peptide quantity
Major Group Top N:	True
Max:	3
Min:	2
Minor Group Quantity:	Sum precursor quantity
Minor Group Top N:	False

DIA Analysis\Workflow	
Method Evaluation:	False
MS2 DeMultiplexing:	Automatic
Profiling Strategy:	iRT Profiling
Carry-over exact Peak Boundaries:	False
Profiling Row Selection:	Minimum Qvalue Row Selection
Qvalue Threshold:	0.001
Profiling Target Selection:	Profile only non-identified Precursors
Identification Criterion:	Qvalue
Threshold:	0.001
Run Limit for directDIA Library:	-1
Unify Peptide Peaks Strategy:	Select corresponding Peak
DIA Analysis\XIC Extraction	
XIC IM Extraction Window:	Dynamic
Correction Factor:	1
XIC RT Extraction Window:	Dynamic
Correction Factor:	1
MS1 Mass Tolerance Strategy:	Dynamic
Correction Factor:	1
MS2 Mass Tolerance Strategy:	Dynamic
Correction Factor:	1
Pulsar Search\Identification	
PSM FDR:	0.01
Peptide FDR:	0.01
Protein Group FDR:	0.01
PTM Localization Filter:	False
Pulsar Search\Modifications	
Max Variable Modifications:	5
Select Modifications:	
Fixed Modifications::	Carbamidomethyl (C)
Variable Modifications: :	Acetyl (Protein N-term), Oxidation (M)
Pulsar Search\Peptides	
Enzymes / Cleavage Rules:	Trypsin/P
Digest Type:	Specific
Max Peptide Length:	52
Min Peptide Length:	7
Missed Cleavages:	2
Toggle N-terminal M:	True
