# Supplementary material for: Zyxin is important for the stability and function of podocytes, especially during mechanical stretch
Source: Commun Biol. 2024 Apr 11;7:446. doi: 10.1038/s42003-024-06125-5 (PMC11009394; doi:10.1038/s42003-024-06125-5)
Supplement: Supplementary file 2 — Description of Additional Supplementary Files [file 42003_2024_6125_MOESM2_ESM.pdf]

Description of Additional Supplementary Files

File name: Supplementary Data 1
Description: The mass spectrometry proteomics data of Zyx-KO podocytes.

File name: Supplementary Data 2
Description: Source data underlying the graphs in the paper.
