# Supplementary material for: Zyxin is important for the stability and function of podocytes, especially during mechanical stretch
Source: Commun Biol. 2024 Apr 11;7:446. doi: 10.1038/s42003-024-06125-5 (PMC11009394; doi:10.1038/s42003-024-06125-5)
Supplement: Supplementary file 5 — Reporting Summary [file 42003_2024_6125_MOESM5_ESM.pdf]

nature portfolio

Corresponding author(s): Felix Kliewe

Last updated by author(s): Feb 28, 2024

Reporting Summary

Nature Portfolio wishes to improve the reproducibility of the work that we publish. This form provides structure for consistency and transparency in reporting. For further information on Nature Portfolio policies, see our Editorial Policies and the Editorial Policy Checklist.

Statistics

For all statistical analyses, confirm that the following items are present in the figure legend, table legend, main text, or Methods section.

n/a	Confirmed	
☐	☒	The exact sample size (n) for each experimental group/condition, given as a discrete number and unit of measurement
☐	☒	A statement on whether measurements were taken from distinct samples or whether the same sample was measured repeatedly
☐	☒	The statistical test(s) used AND whether they are one- or two-sided<br>*Only common tests should be described solely by name; describe more complex techniques in the Methods section.*
☒	☐	A description of all covariates tested
☒	☐	A description of any assumptions or corrections, such as tests of normality and adjustment for multiple comparisons
☐	☒	A full description of the statistical parameters including central tendency (e.g. means) or other basic estimates (e.g. regression coefficient) AND variation (e.g. standard deviation) or associated estimates of uncertainty (e.g. confidence intervals)
☒	☐	For null hypothesis testing, the test statistic (e.g. F, t, r) with confidence intervals, effect sizes, degrees of freedom and P value noted<br>*Give P values as exact values whenever suitable.*
☒	☐	For Bayesian analysis, information on the choice of priors and Markov chain Monte Carlo settings
☒	☐	For hierarchical and complex designs, identification of the appropriate level for tests and full reporting of outcomes
☒	☐	Estimates of effect sizes (e.g. Cohen's d, Pearson's r), indicating how they were calculated

Our web collection on statistics for biologists contains articles on many of the points above.

Software and code

Policy information about availability of computer code

Data collection	Microscopy images were collected with Olympus' FV3000 imaging software.<br>Real-time qPCR were performed with QuantStudio 5 System and data were collected with QuantStudio Design & Analsis Software (v1.5.2).
Data analysis	Statistical analysis were performed with Prism 9.0 (GraphPad). Images were analyzed and measured with ImageJ.

For manuscripts utilizing custom algorithms or software that are central to the research but not yet described in published literature, software must be made available to editors and reviewers. We strongly encourage code deposition in a community repository (e.g. GitHub). See the Nature Portfolio guidelines for submitting code & software for further information.

Data

Policy information about availability of data

All manuscripts must include a data availability statement. This statement should provide the following information, where applicable:

- Accession codes, unique identifiers, or web links for publicly available datasets
- A description of any restrictions on data availability
- For clinical datasets or third party data, please ensure that the statement adheres to our policy

All data supporting the findings of this study are available within the paper and its Supplementary Information and Supplementary Data.
Further information are available from the authors upon reasonable request.

Research involving human participants, their data, or biological material

Policy information about studies with human participants or human data. See also policy information about sex, gender (identity/presentation), and sexual orientation and race, ethnicity and racism.

Reporting on sex and gender	na
Reporting on race, ethnicity, or other socially relevant groupings	na
Population characteristics	na
Recruitment	na
Ethics oversight	na

Note that full information on the approval of the study protocol must also be provided in the manuscript.

Field-specific reporting

Please select the one below that is the best fit for your research. If you are not sure, read the appropriate sections before making your selection.

☒ Life sciences ☐ Behavioural & social sciences ☐ Ecological, evolutionary & environmental sciences

For a reference copy of the document with all sections, see nature.com/documents/nr-reporting-summary-flat.pdf

Life sciences study design

All studies must disclose on these points even when the disclosure is negative.

Sample size	The sample size of each experiment was described in the figure legends. Minimum three samples were used.
Data exclusions	No data were excluded.
Replication	We make at least three repeats of each experiments. The variability was reported in the graphs and figure legends.
Randomization	The experiments were not randomized.
Blinding	The experiments were not blinded.

Reporting for specific materials, systems and methods

We require information from authors about some types of materials, experimental systems and methods used in many studies. Here, indicate whether each material, system or method listed is relevant to your study. If you are not sure if a list item applies to your research, read the appropriate section before selecting a response.

Materials & experimental systems

n/a	Involved in the study	
☐	☒	Antibodies
☐	☒	Eukaryotic cell lines
☒	☐	Palaeontology and archaeology
☐	☒	Animals and other organisms
☒	☐	Clinical data
☒	☐	Dual use research of concern
☒	☐	Plants

Methods

n/a	Involved in the study	
☒	☐	ChIP-seq
☒	☐	Flow cytometry
☒	☐	MRI-based neuroimaging

Antibodies

Antibodies used	The following antibodies were used for immunostaining: anti-zyxin (Z4751, Sigma-Aldrich, St. Louis, MO, USA), anti-zyxin (HPA004835 for human and HPA073497 for mice FFPE material; both from Sigma-Aldrich) anti-paxillin (610051, BD Biosciences), anti-talin (T3287, Sigma-Aldrich), anti-vinculin (V9131, Sigma-Aldrich), anti-fibronectin (ab2413, Abcam), anti-fascin-1 (HPA005723, Sigma-Aldrich) and anti-VASP (HPA005724, Sigma-Aldrich).

	Secondary antibodies: Cy2- or Cy3-conjugated secondary antibodies (Jackson ImmunoResearch Laboratories, West Grove, USA).<br><br>For Western blot we used the following antibodies:<br>anti-zyxin (Z4751, Sigma-Aldrich, St. Louis, MO, USA), anti-paxillin (610051, BD Biosciences), anti-talin (T3287, Sigma Aldrich), anti-vinculin (V9131, Sigma-Aldrich), anti-fibronectin (ab2413, Abcam), anti-fascin-1 (HPA005723, Sigma-Aldrich), anti-VASP (HPA005724, Sigma-Aldrich), anti-filamin A (SAB4500951, Sigma-Aldrich), anti-β actin (sc-47778, Santa Cruz), anti-α-actinin 1 (A5044, Sigma-Aldrich), anti- α-actinin 4 (0042-05, immunoGlobe) and anti-Gapdh (10494-1-AP, Proteintech Group). Secondary Antibodies: HRP–conjugated secondary antibody anti-mouse (SA00001-1, Proteintech Group) or anti-rabbit (SA00001-2, Proteintech Group).<br><br>For histology/tissue staining the following antibodies were used:<br>anti-zyxin (HPA004835 for human and HPA073497 for mice FFPE material; both from Sigma-Aldrich; IF dilution 1:100) and anti-synaptopodin (61094, Progen Biotechnik GmbH, Heidelberg, Germany; IF dilution 1:100)
Validation	Antibody validation is presented in the manufacturers' datasheets.

Eukaryotic cell lines

Policy information about cell lines and Sex and Gender in Research

Cell line source(s)	Conditionally immortalized podocytes (SVI; CLS Cell Line Service GmbH, Eppelheim, Germany) were used and handled as described previously (PMID: 11181788)
Authentication	Authentication by immunostaining and PCR.
Mycoplasma contamination	Cells were regularly tested and reported negative for mycoplasma contamination
Commonly misidentified lines<br>(See ICLAC register)	NA

Animals and other research organisms

Policy information about studies involving animals; ARRIVE guidelines recommended for reporting animal research, and Sex and Gender in Research

Laboratory animals	The Zyx KO mice were kindly provided by the group of M. Hecker (Heidelberg; Germany). The strain was originally designed by M. Beckerle (Utah; USA), but backcrossed more than 10-times onto the C57BL/6J background. As wildtype control age-matched C57BL/6J mice were used. Experiments were done with 6-month-old and 12-month-old male and female mice.
Wild animals	This study does not involve wild animals.
Reporting on sex	Experiments were done with male and female mice.
Field-collected samples	This study does not involve samples collected from the field.
Ethics oversight	All studies were carried out in strict accordance with regulations in Germany regarding the use of laboratory animals and were approved by the regional councils.

Note that full information on the approval of the study protocol must also be provided in the manuscript.

Plants

Seed stocks	na
Novel plant genotypes	na
Authentication	na
